# Assembly and annotation of the mitochondrial minicircle genome of a differentiation-competent strain of *Trypanosoma brucei*

**DOI:** 10.1093/nar/gkz928

**Published:** 2019-10-30

**Authors:** Sinclair Cooper, Elizabeth S Wadsworth, Torsten Ochsenreiter, Alasdair Ivens, Nicholas J Savill, Achim Schnaufer

**Affiliations:** 1 Institute of Immunology & Infection Research, University of Edinburgh, Edinburgh, Scotland EH9 3FL, UK; 2 Institute of Cell Biology, University of Bern, CH-3012 Bern, Switzerland

## Abstract

Kinetoplastids are protists defined by one of the most complex mitochondrial genomes in nature, the kinetoplast. In the sleeping sickness parasite *Trypanosoma brucei*, the kinetoplast is a chain mail-like network of two types of interlocked DNA molecules: a few dozen ∼23-kb maxicircles (homologs of the mitochondrial genome of other eukaryotes) and thousands of ∼1-kb minicircles. Maxicircles encode components of respiratory chain complexes and the mitoribosome. Several maxicircle-encoded mRNAs undergo extensive post-transcriptional RNA editing via addition and deletion of uridines. The process is mediated by hundreds of species of minicircle-encoded guide RNAs (gRNAs), but the precise number of minicircle classes and gRNA genes was unknown. Here we present the first essentially complete assembly and annotation of the kinetoplast genome of *T. brucei*. We have identified 391 minicircles, encoding not only ∼930 predicted ‘canonical’ gRNA genes that cover nearly all known editing events (accessible via the web at http://hank.bio.ed.ac.uk), but also ∼370 ‘non-canonical’ gRNA genes of unknown function. Small RNA transcriptome data confirmed expression of the majority of both categories of gRNAs. Finally, we have used our data set to refine definitions for minicircle structure and to explore dynamics of minicircle copy numbers.

## INTRODUCTION

Single-cellular flagellates of the order *Kinetoplastida* include many important parasites of humans and their livestock (1,2) and are characterized by an eponymous structure within their single mitochondrion, the kinetoplast (3,4). The kinetoplast represents the mitochondrial DNA of these organisms (also called kinetoplast DNA, or kDNA) and is typically, but not always, organised as a massive, disk-shaped network of interlinked DNA circles (5).

In *Trypanosoma brucei*, the causative agent of human African trypanosomiasis (HAT, or sleeping sickness), kDNA is particularly complex. It consists of two types of molecules: dozens of essentially identical copies of a ∼23-kb maxicircle, and 5000–10 000 minicircle molecules, which are ∼1-kb in size and highly diverse. The maxicircle is the equivalent of mitochondrial DNA in other eukaryotes and encodes 18 protein components of the mitochondrial respiratory chain and the mitoribosome, and two mitoribosomal RNAs (6). However, 12 of the protein-coding genes are ‘cryptogenes’—their mRNAs require post-transcriptional RNA editing by insertion and, less frequently, deletion of uridylate (U) residues (7,8). Nine of these mRNAs require particularly extensive ‘pan-editing’ via the insertion of hundreds and the deletion of dozens of Us per transcript. The precise sites of U insertion and deletion are specified by ‘guide RNAs’ (gRNAs), short transcripts of typically 40–60 nt in lengths that, from 5′ to 3′, are characterized by the following features: (i) a 5′ triphosphate, marking them as primary transcripts, (ii) a semi-conserved 5′ transcript initiation sequence that has been reported to conform to a 5′-RYAYA consensus (9), (iii) an ‘anchor’ sequence (which can overlap with the initiation sequence) that is crucial for recognising the cognate pre-edited mRNA via Watson–Crick base-pairing, (iv) the ‘information region’, which is complementary to a particular stretch of edited mRNA (but involves non-Watson-Crick G:U base pairs) and (v) a 3′ oligo(U) tail that is added post-transcriptionally and that has been suggested to stabilise the gRNA-mRNA interaction (10).

The four core enzymatic activities involved in trypanosome U-insertion/deletion editing - endonucleolytic mRNA cleavage, U addition to or U removal from the 3′ end of the 5′ cleavage product, RNA ligation - are contained within a ∼20S ‘RNA editing core complex’ (RECC), but other multi-subunit complexes, dynamically interacting with the RECC and with each other to form a ‘holo-editosome’, are required as well (reviewed in (8,11)). The precise order of U insertion/deletion events within an editing ‘block’ (the region decoded by a single gRNA’s information region) that results in perfect or near-perfect alignment between a gRNA’s information region and the cognate mRNA is unclear (reviewed in (12)), but may involve cycles of editing and progressive realignment of gRNA and mRNA until the thermodynamically most stable structure is achieved (13). With the exception of the cytochrome oxidase subunit II mRNA (COX2, see below), sequential involvement of multiple gRNAs in a 3′ to 5′ direction along an mRNA is required for complete editing: all gRNAs, except the initiating one(s), depend on action of the prior gRNA(s) to generate the recognition sequence for the anchor.

Application of deep sequencing methodology to the study of steady-state transcript populations of pan-edited mRNAs has revealed an extraordinary complexity, with the vast majority of transcripts representing partially edited molecules with unedited 5′ regions, fully edited 3′ regions, and middle regions in various states of editing (14–18). An important open question concerns the role of transcripts with ‘non-canonical’ edited sequences that have been detected: do at least some of these transcripts represent functional mRNAs that encode alternative editing products, as has been suggested (19), or are they simply by-products of an error-prone process? Thus, our present knowledge regarding functional, fully edited mRNA sequences is incomplete.

Intricately linked to the question of alternative editing is the desire to know the entire set of gRNAs present in a cell. With the exception of two maxicircle-encoded gRNAs, *T. brucei* gRNAs are encoded by minicircles, but the true complexity of the minicircle population in this species is unknown. Based on reassociation kinetics, it has been estimated that there are 200–250 different minicircle ‘classes’ or types in this species (20,21), but repertoires reported for individual strains are incomplete (22,23). Estimates of minicircle sequence diversity are complicated by the lack of a precise definition of the term ‘minicircle class’. Some authors have argued that a class is defined by the encoded set of gRNAs (typically 3–5 gRNA genes are found per minicircle (9,22)), but cases have been reported where minicircles share the same gRNA genes in the same order, despite only ∼65% of overall sequence conservation (24). Other authors have used a more stringent cut-off of >95% overall sequence identify to assign minicircles to the same class (23). Recent studies have used deep sequencing of short mitochondrial RNAs in *T. brucei* to identify sets of gRNAs that provide nearly complete coverage of the 12 known edited mRNAs (25,26). Although these studies were extremely valuable, for example by dramatically expanding the repertoire of known gRNAs, they faced the challenges of identifying gRNAs that were produced from the same vs. a closely related gene and of distinguishing genuine gRNAs from other sequences. Because the gRNA identification strategy in these studies relied on matches to known edited sequence they also had limited capacity to identify novel ‘non-canonical’ gRNAs that might specify hitherto unknown editing events.

Here, we report assembly and annotation of the first close-to-complete kDNA genome for any strain of *T. brucei*. We have assembled 391 complete, circular sequences that likely represent nearly all minicircles that were present in the parasite population being studied. We used complementarity to known edited mRNA sequences and conserved features such as nucleotide bias and inverted repeats to predict gRNA genes in the assembled minicircles, and we complemented these predictions with the experimentally determined small RNA transcriptome from the same cell line. Importantly, the parasite strain we chose for kDNA and small RNA isolation is capable of differentiating from the lifecycle stage that infects the mammalian bloodstream to the stage that infects the midgut of the tsetse fly vector, two stages that require expression of distinct subsets of kDNA-encoded genes. With this approach we identified more than 900 canonical gRNAs that cover nearly all known edited sequences. We also identified ∼370 putative non-canonical gRNAs. Finally, we have used our dataset to refine the definitions for minicircle structure and to explore the dynamics of minicircle copy numbers.

## MATERIALS AND METHODS

### Trypanosome cell culture and differentiation

The generation of the two cell lines used for this study (Table 1) was described previously (27). Briefly, bloodstream form (BSF) parasites of the pleomorphic (i.e. differentiation competent) *T. brucei* strain EATRO 1125, cell line AnTat1.1 90:13 (28), were transfected with a construct for *in situ* replacement of F_1_F_O_-subunit γ (systematic ID Tb927.10.180) with either a wild type (WT) allele of the same gene or with an allele with the L262P point mutation, which renders BSF *T. brucei* independent of kDNA (29). The rationale for using these cell lines was to simultaneously study the effects of kDNA-independence on kDNA composition, which will be reported elsewhere. Cells were maintained in HMI-9 medium (30) supplemented with 10% FBS at a cell density no greater than 10^6^ cells/ml. In order to generate a procyclic from (PCF) equivalent of the WT BSF cell line, a mouse was infected with WT BSF cells, and blood with parasites was collected at peak stumpy form density, as described (27). Mouse blood was then added to a flask containing 6 mM *cis*-aconitate in HMI-9 (supplemented with 10% FBS) and incubated at 37°C for 24 h. After the incubation period, the mouse blood had coagulated and settled to the bottom of the flask. The cells were then collected from the top layer, centrifuged (3000g for 5 min), washed with SDM-80 medium (31) and maintained in SDM-80 at 27°C at a cell density no greater that 1 × 10^7^ cells per ml.

**Table 1.** Kinetoplast DNA samples and sequencing reads used in this work

Sample ID	Description	Total number of reads (before QC)	CSB-3 containing reads (% of total reads)	% of CSB-3 reads mapped to nuclear genome	% of CSB-3 reads mapped to minicircle assembly
T0_WT_A	*T. brucei* ATPγ/Δatpγ::atpγWT, starting culture, total DNA isolation	7 578 986	158 721 (2.09)	0.26	96.81
T0_WT_B	*T. brucei* ATPγ/Δatpγ::atpγWT, starting culture, kDNA isolation; *T. brucei* ATPγ/Δatpγ::atpγL262P (kDNA^0^), total DNA isolation	8 704 684	364 084 (4.18)	0.33	91.43
T1_WT_A	*T. brucei* ATPγ/Δatpγ::atpγWT, after ∼40 generations of *in vitro* culture (2 weeks), kDNA isolation	12 471 650	2 634 234 (21.12)	0.00	98.60
T1_WT_B	*T. brucei* ATPγ/Δatpγ::atpγWT, after ∼40 generations of *in vitro* culture (2 weeks), kDNA isolation	8 936 800	1 834 928 (20.53)	0.00	98.65
T2_WT_A	*T. brucei* ATPγ/Δatpγ::atpγWT, after ∼250 generations of *in vitro* culture (12 weeks), kDNA isolation	6 605 380	1 487 120 (22.51)	0.00	98.52
T2_WT_B	*T. brucei* ATPγ/Δatpγ::atpγWT, after ∼250 generations of *in vitro* culture (12 weeks), kDNA isolation	6 358 610	1 274 856 (20.05)	0.00	98.68
T0_L262P_A	*T. brucei* ATPγ/Δatpγ::atpγL262P, starting culture, total DNA isolation	7 284 100	43 383 (0.60)	1.07	92.58
T0_L262P_B	*T. brucei* ATPγ/Δatpγ::atpγL262P, starting culture, kDNA isolation; *T. brucei* ATPγ/Δatpγ::atpγL262P (kDNA^0^), total DNA isolation	8 121 310	134 012 (1.65)	0.89	91.04

Notes: See Supplementary Figure S1 for a timeline for these samples. Cell lines are based on parental cell line *T. brucei* EATRO 1125 AnTat 1.1 90:13 (28). One allele of the F_1_F_O_-ATPase γ subunit had been replaced with either a WT allele (atpγWT) or a version with the L262P mutation (atpγL262P) (27). We prepared DNA for Illumina MiSeq sequencing (300-bp paired-end reads) either directly from whole cell lysates or after enriching for kDNA (32). For samples T0_WT_B and T0_L262P_B, we had pooled kDNA preparations with total DNA from akinetoplastic (kDNA^0^) cells that had been obtained by ethidium bromide treatment of a cell line expressing an F_1_-ATPase γ subunit with the L262P mutation (29). The purpose of this step was to meet minimum input DNA requirements for Illumina TruSeq DNA library preparation for these two samples. For other kDNA samples the Illumina TruSeq Nano Kit was used, which facilitates library preparation from lower amounts of DNA. As indication for kDNA coverage after sequencing, for each sample we determined the number and percentage of reads that contain the conserved CSB-3 minicircle motif, 5′-GGGGTTGGTGTA-3′ (42). We also determined the percentage of CSB-3 containing reads that mapped to the *T. brucei* TREU927 reference genome (34), version 5.2 (downloaded from www.TriTrypDB.org), and to the 391 assembled minicircles.

### Purification of kDNA and total DNA

Kinetoplast DNA was essentially purified as described (32), using the following modifications based on helpful advice from Michele Klingbeil. Parasites (3 × 10^8^ cells for a typical preparation) were harvested from *in vitro* culture by centrifugation, washed in PBS-G (137 mM NaCl, 2.7 mM KCl, 10 mM Na_2_HPO_4_, 1.8 mM KH_2_PO_4_, 55 mM glucose, pH 7.4), and resuspended in 1 ml NET-100 solution (100 mM NaCl, 100 mM EDTA, 10 mM Tris–HCl, pH8.0). The samples were then centrifuged for 5 minutes at 3000 rpm and the supernatant discarded. Cells were lysed by adding 870 μl NET-100, 10 μl Proteinase K (20 mg/ml; Invitrogen) and 100 μl of 10% (w/v) SDS solution before being passed through a 23 gauge needle twice. Samples were then incubated at 56°C overnight and subsequently treated with RNase A (10 μl of 10 mg/ml) at 37°C for 15 min. Each sample was split into two 500 μl volumes, loaded onto 700 μl of a 20% sucrose cushion and centrifuged at 20 000 g for 60 minutes. Most of the supernatant was discarded, leaving behind ∼50 μl residual volume which was then resuspended in 250 μl TE buffer (10 mM Tris–HCl pH 8.0, 1 mM EDTA). The two samples were combined and loaded onto a second 700-μl 20% sucrose cushion. The samples were centrifuged (20 000 g, 60 min), and kDNA purified from the bottom fraction using a standard ethanol precipitation method and dissolved in H_2_O.

For total DNA preparations, DNA was purified using a phenol-chloroform based method as we found that commercial, column based purification methods resulted in loss of kDNA. Briefly, one volume of phenol:chloroform:isoamyl alcohol (25:24:1) was added to a cell pellet (3 × 10^8^ cells for a typical preparation) and agitated with a vortex for 20 s. Samples were centrifuged at 16 000 g for 5 minutes, the upper aqueous phase removed, and DNA purified using by ethanol precipitation.

### Sequencing of kDNA, quality control of reads, and initial processing

In order to generate a reference quality minicircle assembly, multiple samples were pooled to ensure good representation of even minor classes (Table 1). Libraries were constructed from purified kDNA using the TruSeq Nano DNA Sample Prep Kit or from kDNA mixed with total DNA from a cell line lacking kDNA (serving as ‘carrier’) using the Illumina TruSeq DNA Sample Prep Kit (see Table 1). In both cases, DNA was fragmented to generate ∼550 bp inserts and sequenced to generate paired-end Illumina MiSeq 300-bp reads (Edinburgh Genomics). Reads were quality checked and trimmed using fastqc (http://www.bioinformatics.babraham.ac.uk/projects/fastqc/) and Trimmomatic (33) (bases below Q15 were removed). Reads corresponding to the nuclear megabase-sized chromosomes were removed by alignment to the published *T. brucei* TREU 927 genome (34) (v5.2; obtained from http://www.genedb.org/) with bowtie2 (35), using default parameters.

Scripts used for processing sequencing data in this study are available at https://github.com/nicksavill/kDNA-annotation. This includes scripts for general bioinformatics tasks as well as specific programmes. Each script includes all the information required for appropriate use.

### Maxicircle assembly

Contigs were assembled using Velvet (36) and a 16.4-kb fragment that includes all known maxicircle genes was identified by similarity searching using ublast (37) against the published, 23.0-kb maxicircle sequence from *T. brucei* strain Lister 427 (M94286.1). This contig was missing the variable region (38), presumably because the repeat nature of this region makes it difficult to assemble using conventional short reads. An attempt to close the gap using a PCR approach was not successful. Maxicircle gene annotations were transferred manually from M94286.1 and the 16.4-kb EATRO 1125 maxicircle fragment was submitted to GenBank (accession number MK584625).

### Minicircle assembly and identification of conserved motifs

Various assemblies were performed using Velvet (36) with a range of kmer values and coverage cut-off values (coverage cut-off was deemed to be important because we expected large variations in the copy number for each of the classes of minicircle (39)). It should be noted that there is no generally accepted or applicable coverage cut-off for minicircle assembly, and this must be optimised for each data set, reflecting differences in library preparation, cell lines, kDNA coverage and kDNA complexity. Two alternative assemblies were selected from a range of assemblies generated: one had a high number of diverse but incomplete contigs whilst the other had a smaller number of complete (∼1 kb) contigs. The first assembly was performed with a coverage cut-off of 10 to elucidate low coverage contigs and the second was performed with a coverage cut-off of 60. These assemblies produced 565 and 452 contigs, respectively. The two assemblies were merged using the CAP3 assembly algorithm (40) using the default parameters.

An artefact of the way in which assembly algorithms like Velvet handle Illumina type short read data from circular molecules is that fully assembled contigs have their ends duplicated. Searching for the presence of such duplicated ends was used to identify contigs derived from circular sources with a ‘circularity test’ (similar in approach to (41)). Contigs were sliced in half prior to passing the halves through the CAP3 assembly algorithm; a bash batch processing script ensured that each pair of halves was processed separately (avoiding the possibility of any chimeric contigs being re-assembled if, for example, the contigs happened to be sliced in a conserved region). Minicircles sequences that passed the circularity test were checked for the presence of the conserved sequence block 3 (CSB-3) dodecamer (GGGGTTGGTGTA), also known as the universal minicircle sequence (42), using a custom Python script. Unexpectedly, three minicircles had a slight variation of this sequence, GGGGTTGATGTA. Duplicate sequences that had >95% identity (not counting the conserved region) were removed. This approach resulted in the assembly of 344 complete minicircles. In a final round of assembly, reads not mapping to these 344 minicircles were assembled with Geneious (Biomatters Ltd., New Zealand), using default settings for assembly of circular contigs. Contigs lacking the CSB-3 dodecamer or sharing more than 95% identity were again removed. This process resulted in the identification of a total of 391 minicircle sequences.

Imperfect 18-bp inverted repeat sequences, originally identified as semi-conserved minicircle features by Jasmer and Stuart (43) and later found to define most gRNA gene cassettes (9), were identified in two steps. First, minicircles were searched by regular expression pattern matching for the semi-conserved upstream (forward) sequence TAATA[GA]AT and the semi-conserved downstream (reverse) sequence AT[TC]TATTA separated by 100–150 bp. If a forward sequence was found but not a reverse sequence, the regular expression for the reverse sequence was shortened to TATTA and a new search made 100–150 bp downstream from the forward sequence. If a reverse sequence was found but not a forward sequence, the regular expression for the forward sequence was shortened to TAATA and a new search made 100–150 bp upstream from the reverse sequence. This step identified 1290 pairs of sequences. In order to accurately identify all 18-bp inverted sequences we next used a nucleotide bias scoring method. The frequencies of nucleotides at each position from –20 nt upstream to +20 nt downstream of the 1290 forward sequences and the 1290 reverse sequences were obtained from minicircle sequences. This procedure resulted in a forward nucleotide frequency matrix $f_{i,j}^{( f )}$, where $i \in \{ {{\rm{A,\ C,\ G,\ T}}} \}$ is nucleotide and $j \in [ { - 20,\ 20} ]$ is position, and a reverse nucleotide frequency matrix $f_{i,j}^{( r )}$. The nucleotide frequency matrices were then used to generate forward and reverse scoring vectors ${S^{( f )}}$ and ${S^{( r )}}$, respectively, along each minicircle. The forward score $S_k^{( f )}$, at position k on a minicircle, is the sum of the nucleotide frequencies from –20 nt upstream to +20 nt downstream of position $k$, i.e.,

$$\begin{equation*}\ S_k^{\left( f \right)} = \mathop \sum \limits_{j = - 20}^{20} f_{{n_{k + j}},j}^{\left( f \right)}\ \ {\rm{for}}\ k\ = \ 21, \ldots ,N - 20\end{equation*}$$

where $N$ is the length of the minicircle, and ${n_{k + j}}$ is the nucleotide at position $k + j$ on the minicircle. The reverse score at position k on a minicircle is given by

$$\begin{equation*}\ S_k^{\left( r \right)} = \mathop \sum \limits_{j = - 20}^{20} f_{{n_{k + j}},j}^{\left( r \right)}\ \ {\rm{for}}\ k\ = \ 21, \ldots ,N - 20\end{equation*}$$

Pairs of peaks in the forward and reverse scores between 80 and 150 bp apart were identified as 18-bp inverted repeats. This step identified an additional five pairs of 18-bp inverted repeats bringing the total to 1295.

### Validation of minicircles sequences by PCR

To confirm fidelity of the assembly process, 13 minicircles were selected at random for validation by PCR. A pair of specific primers was designed for each minicircle using OligoPicker (44):

mO_007: 5′-TGCTTTTTCCTGTTTATTCTGAGAT / 5′-TATCCACAGAAATAACACTACTACT

mO_031: 5′-TCAGTATTCTTATCACCTCCATTAT / 5′-AAATCAGTAGGAAAAGTAAGGTGTA

mO_045: 5′-ACTCTGCATAACTTCTACTAACAAA / 5′-TTTTGAGTTTGATAATTGGATGGGT

mO_135: 5′-ATTACAAAAATCCCGAAAAAGGAGT / 5′-TTATGGTTTAGAGATTAGTAAGTGT

mO_138: 5′-TAAGAGGGACAGAGTAATATAAGTT / 5′-GATTAGCACTTTAACTCTGCAATTA

mO_143: 5′-TAAATCCAAAAATTCCCTGAAACTC / 5′-TACTTTAGATGGTAGATGAGTAGTT

mO_180: 5′-GGTTTTTCTCACTTATTTGGCTTTA / 5′-TGTAATTCTCTACCATATACTTCAC

mO_197: 5′-GGTTTTGAGTTTATCTAGGTTTTGA / 5′-TTTTCTTTCTTTATCTTGAAGCCCT

mO_230: 5′-AAAATTCCCCAAAATTCCCCAAAAT / 5′-TGTAGATGGATGTGAATGGAATTTT

mO_248: 5′-GTGTTATTGAATGTCTCAAAGGTAA / 5′-TTTATCTTTCTAGAGACCTCGAAAA

mO_307: 5′-TATTTTATTCCTTCACCCTTGAATA / 5′-CTGATTTATAAGTTGGGAATAGAAG

mO_335: 5′-GGTTTTTCTGGAATTTTCAGCTTAT / 5′-CTTTAGAGTCAAACTTAATAACCCT

mO_347: 5′-CACACGGTTTTTTCACATTTATTCA / 5′-AATTCCTAATAGCAGATTCTTCTTC

A 25*-*μl reaction was prepared for each primer set containing 1X GoTaq PCR buffer (Promega), 200 μM dNTP (Promega), 1.5 mM MgCl_2_, 1 μM forward and reverse primers, 1 unit GoTaq polymerase (Promega), and 20–60 ng of purified total DNA (*T. brucei* EATRO 1125 AnTat1.1 cell line). Cycle conditions were: 94°C, 5 min, followed by 30 cycles of 94°C for 2 min, 50°C for 1 min and 72°C for 1.5 min, followed by a final extension step 72°C for 8 min. Products were analysed by agarose gel electrophoresis and ethidium bromide staining. Amplified DNA was directly purified from the remaining PCR reactions (PCR clean up kit, Qiagen) and either cloned into the pGEM-T-Easy vector (Promega) and Sanger sequenced, or directly Sanger sequenced (Edinburgh Genomics).

### Prediction of canonical gRNA genes

In order to address strain-specific differences in edited mRNA sequences, A, C and G residues in published sequences were corrected based on the assembled EATRO 1125 maxicircle sequence (see above; GenBank submission MK584625). Next, Illumina reads available from an EATRO 1125 whole-cell RNA sequencing project (45) were aligned to these edited sequences using CLC Genomics Workbench v4.9 and the alignments carefully inspected for indications of potential differences from the published editing patterns. These transcriptome data had incomplete coverage of the mitochondrial mRNAs, but still offered useful guidance. Most importantly, these data suggested two alternative versions for A6 and ND8 mRNAs (see below). Canonical gRNAs that could be aligned to the collection of edited sequences were then predicted in three steps.

Step 1. Coding and template strands of assembled maxicircle and minicircles were aligned (not permitting gaps) to edited mRNA to predict canonical gRNAs inside and outside of cassettes. Each circle's coding and template strands were split into 120-nt fragments with each fragment overlapping by 60 nt. Given that gRNA genes are about 40 nt long, an overlap of 60 nt was deemed sufficient to capture all gRNAs. Each fragment was then aligned to the edited mRNA sequences. All alignments which included at least one U insertion or deletion, which contained a 5′ anchor of at least six Watson–Crick basepairs and which contained a minimum of 24 matches (Watson–Crick or G–U basepairs) with no contiguous mismatches were recorded. Duplicates were discarded. By recording all alignments, and not just a highest scoring alignment (such as is done in Smith–Waterman alignments), gRNA genes could be discovered that have the potential to edit multiple mRNAs. Gaps in alignments were not allowed in this step due to prohibitively long computer processing times.

Step 2. Coding and template strands of cassettes defined by 18-bp inverted repeats were aligned to edited mRNA, this time permitting gaps. This step recovered non-gapped canonical gRNAs inside cassettes, extended non-gapped canonical gRNAs, usually at their 3′ ends, and discovered new gapped canonical gRNAs in cassettes in which no canonical gRNAs were found in step 1. Here, no fragmentation step was necessary before each cassette's coding and template strands were aligned to the edited mRNAs. All unique alignments which included at least one U insertion or deletion, which contained a 5′ anchor of at least six Watson–Crick basepairs, and which contained 24–65 matches with no gaps greater than 2 basepairs were recorded. This produced a very large number of potential alignments for each cassette. Each alignment was ranked on the following criteria:

- Rank 1: at least 25 contiguous matches including the anchor
- Rank 2: at least 25 contiguous matches
- Rank 3: two adjacent regions of contiguous matches, totalling at least 25 matches
- Rank 4: three adjacent regions of contiguous matches, totalling at least 25 matches

The highest ranked alignments in each cassette, of which there were always more than one, were kept and all lower ranking alignments were discarded. These remaining top-ranked alignments were further accepted on the following characteristics:

- 1. the longest contiguous number of matches
- 2. the most number of matches
- 3. the lowest number of mismatches and gaps divided by the alignment length

Step 3. For our gapped gRNA dataset, gRNAs in the same cassette that edited the same region of mRNA found in steps 1 and 2 were combined to obtain the longest gRNA. For our non-gapped dataset, any gapped version of a non-gapped gRNA was ignored, and any gapped gRNA without a corresponding non-gapped version was trimmed. If the resulting gRNA was at least 25 nt long and formed a 6-bp Watson–Crick anchor with its mRNA, it was retained. Probable false positives were removed after careful visual inspection. Removal criteria were somewhat subjective but generally removed many gRNAs around 25–29 nt with gaps, mismatches or incorrect positioning. Such likely false positive gRNAs included the following: (i) gRNAs in cassettes with multiple gRNAs that are 5 nt shorter than the longest gRNA in that cassette; (ii) non-expressed gRNAs not in cassettes shorter than 30 nt; (iii) gRNAs that had poor overlap with small RNAs expressed in the same cassette (see below); (iv) gRNAs that overlap an inverted repeat.

### Prediction of non-canonical gRNA genes

Putative non-canonical, coding strand gRNAs within cassettes were predicted as follows. Guide RNAs found in cassettes in Step 1 above were aligned at their 5′ ends. The frequencies of nucleotides at each position from -80 nt upstream to +80 nt downstream of the aligned gRNA genes were obtained from minicircle sequences. The same procedure was carried out with the gRNA genes aligned at their 3′ ends. This procedure resulted in an upstream nucleotide frequency matrix $f_{i,j}^{( u )}$, where $i \in \{ {{\rm{A,\ C,\ G,\ T}}} \}$ was nucleotide and $j \in [ { - 80,\ 80} ]$ was position, and a downstream nucleotide frequency matrix $f_{i,j}^{( d )}$. The nucleotide frequency matrices were then used to generate upstream and downstream scoring vectors ${S^{( u )}}$ and ${S^{( d )}}$, respectively, along each minicircle. The upstream score $S_k^{( u )}$, at position $k$ on a minicircle, is the sum of the nucleotide frequencies, i.e.,

$$\begin{equation*}\ S_k^{\left( u \right)} = \mathop \sum \limits_{j = - 80}^{80} f_{{n_{k + j}},j}^{\left( u \right)}\ \ {\rm{for}}\ k\ = \ 81, \ldots ,N - 80\end{equation*}$$

where $N$ is the length of the minicircle and ${n_{k + j}}$ is the nucleotide at position $k + j$ on the minicircle. The downstream score at position k on a minicircle is given by

$$\begin{equation*}\ S_k^{\left( d \right)} = \mathop \sum \limits_{j = - 80}^{80} f_{{n_{k + j}},j}^{\left( d \right)}\ \ {\rm{for}}\ k\ = \ 81, \ldots ,N - 80\end{equation*}$$

Peaks in the upstream and downstream score vectors correspond to the start and end of gRNAs (see Figure 2B) and were chosen to predict putative non-canonical gRNAs. The upstream and downstream scores are noisy, preventing a precise positioning of non-canonical gRNAs. To overcome this, the two scoring vectors were added together but offset by 40 nt (which is roughly the length of the encoded gRNAs). This ensured that two close peaks representing the start and end of a gRNA constructively interfere, and single anomalous peaks are disregarded. The resulting combined scoring vector and its derivative were smoothed using the Savitzky–Golay filter (window size of 31, order 5). Peaks were identified where the derivative became negative. Peaks above a certain cut-off were identified as the start of a non-canonical gRNA. The cut-off peak size was determined by finding the minimum peak size of all canonical gRNAs, which was 0.685.

### Small RNA preparation, sequencing, quality control, and initial processing

Crude mitochondrial fractions were prepared from ∼10^9^ BSF cells of *T. brucei* EATRO 1125 AnTat1.1 90.13 (WT ATPase subunit γ replacement), and from the same number of PCF cells after differentiation (see above), by hypotonic lysis and differential centrifugation as described previously (46). Total RNA was isolated using Trizol reagent (Thermo Fisher), and the small fraction of <200 bp was then size selected using the PureLink miRNA isolation kit (Thermo Fisher). Isolated small RNAs were then treated with RNA 5′ polyphosphatase (Epicentre), as per the manufacturer's instructions, to degrade all 5′ tri-phosphates, present at the 5′ ends of gRNAs (47), to mono-phosphates. After phenol-chloroform extraction, Illumina small RNA libraries were generated following the manufacturer's instructions and sequenced using an Illumina HiSeq platform to generate 125-bp paired reads (Centre for Genomics Research, University of Liverpool).

Barcode separated libraries were quality checked using fastqc as above and adapter-trimmed with cutadapt version 1.9.1 (48). As the insert length for gRNAs is expected to be shorter than the length of the reads, Illumina adapters were searched for from both the 5′ and 3′ end of both the forward and reverse read. Untrimmed reads and those that were shorter than 15 bp after adapter trimming were also discarded. If both the forward and reverse reads contained the 5′ and 3′ adapter they were merged using PEAR (49) and mapped to the nuclear megabase-sized chromosomes using bowtie2 as described above. Reads that mapped to the nuclear genome were discarded at this stage.

### Minicircle expression analysis

Small RNA reads not aligning to the nuclear genome were mapped to the *T. brucei* EATRO 1125 maxicircle and to the 391 minicircles using CLC Genomics Workbench v4.9 (parameters: similarity = 0.95; length fraction = 0.5; global alignment = no; match mode = random; insertion cost = 3; deletion cost = 3; mismatch cost = 2). Small RNAs overlapping with predicted canonical or non-canonical gRNA genes were annotated manually by visually inspecting read coverage and subjectively defining consensus start and end points for the templated parts of the transcripts.

To identify ‘expressed’ gRNA genes, alignment bam files were filtered to retain only sense (coding, or forward strand) or antisense (template, or reverse strand) alignments. Depth values were extracted from the re-indexed bam files using samtools v1.3 (50). During plotting of the data, predicted peaks within each minicircle sequence were identified as regions where read depth exceeded a subjectively assigned percentage of total read depth (0.025%) for that minicircle in the sample of interest.

### Identification of gRNA anchor regions

For each canonical gRNA we identified its maximum anchor as the longest, 5′-most stretch of Watson–Crick basepairs. An anchor is considered an extender if there are any insertions or deletions in its first 6 bp, or an initiator if there are no insertions or deletions in its first 6 bp.

### Coverage statistics

For the purpose of obtaining coverage statistics we considered a canonical gRNA’s anchor region as the 5′-most stretch of 6 Watson–Crick basepairs and its information region as the rest of the gRNA including any mismatches. We considered a U insertion or deletion to be covered on an mRNA if an information region of a gRNA aligned with that insertion or deletion. The number of missing gRNAs was obtained by assuming that the standard information region of a gRNA is 34 bp long (40 bp minus the 6-bp anchor). We counted the minimum number of such gRNAs necessary to cover U insertions and deletions not covered by identified canonical gRNAs.

## RESULTS

### Minicircle assembly for *T. brucei* EATRO 1125

We isolated total genomic DNA or kDNA from eight separate cultures of *T. brucei* strain EATRO 1125 (cell line AnTat 1.1 90:13 (28)) and sequenced the content using Illumina MiSeq 300-bp paired-end technology (Table 1; Supplementary Figure S1 shows a timeline for these samples). As an indication for kDNA coverage in these samples, we determined the percentage of reads containing conserved sequence block 3 (CSB-3) also known as the universal minicircle sequence (42). As expected, reads with the CSB-3 sequence were much more frequent in samples enriched for kDNA than in total DNA samples (20–23% versus 0.6–4.2%; Table 1).

For assembly of minicircles we combined reads from all samples to increase the chance of capturing minor sequence classes and used a combination of computational tools (see Materials and Methods for details). We obtained a total of 391 distinct circular contigs (we considered any minicircle as distinct that had less than 95% sequence identity to any other minicircle in the assembly, discounting 100 nt of the conserved region). These contigs ranged in size from 963 to 1084 bp, with a peak at ∼1000 bp (Supplementary Figure S2A), as expected for *T. brucei* minicircles (22,51). Mapping all reads back to the 391 contigs achieved complete coverage for all of them, with average coverage ranging from ∼5-fold to ∼100 000-fold (Supplementary Table S1). All contigs contained a single copy of the previously described conserved region of ∼120 bp that contains the origin of replication and three conserved sequence blocks, CSB-1, CSB-2 and CSB-3 (Supplementary Figure S2B) (3,22,42,51). CSB-1 (GGGCGTGCA) was 100% conserved among all 391 minicircles, confirming earlier observations (22,42). In an earlier analysis of 25 *T. brucei* minicircles, two groups had been identified, based on an ‘8-nt substitution’ immediately downstream of CSB-1 (22). While these two sequence variations represent the majority of the minicircles identified here, we found three additional groups (Supplementary Figure S2C). CSB-2 and CSB-3 showed some variability around the consensus reported previously (Supplementary Figure S2B, D). For CSB-3 this was unexpected, as the dodecamer GGGGTTGGTGTA was reported to be universally conserved (42). Nonetheless, careful inspection of the sequencing reads aligning to mO_178, mO_200 and mO_243 confirmed that these three minicircles had a slightly modified CSB-3 sequence, GGGGTTGATGTA.

Also conserved among minicircles was a region immediately upstream of CSB-1, characterised by five A-tracts, each ∼5 bp long and positioned roughly in phase with the helical repeat (Supplementary Figure S2E). These sequence characteristics cause a bend in the minicircle DNA (52) that has been suggested to aid organisation of minicircles into the kDNA structure (3).

To validate the minicircle assembly we used a PCR approach. Out of 13 randomly chosen minicircles, 12 could readily be amplified with specific primers (Supplementary Figure S3), and DNA sequencing confirmed that the PCR products matched the predicted sequences.

To evaluate how completely our assembly represents the minicircle population present in the cultures used for DNA isolation, we mapped all Illumina reads back to the 391 minicircles (Table 1). For the reads for the four preparations enriched for kDNA (which contributed ∼92% of all CSB-3-containing reads), consistently more than 98% of reads containing the CSB-3 sequence mapped to the minicircle assembly. We conclude that our assembly represents a nearly complete representation of the minicircle classes present in the cultures used for DNA isolation. If there are minicircle classes missing from this assembly, they were most likely present at very low frequency.

### Estimation of minicircle copy numbers and network size

**Figure 1.**
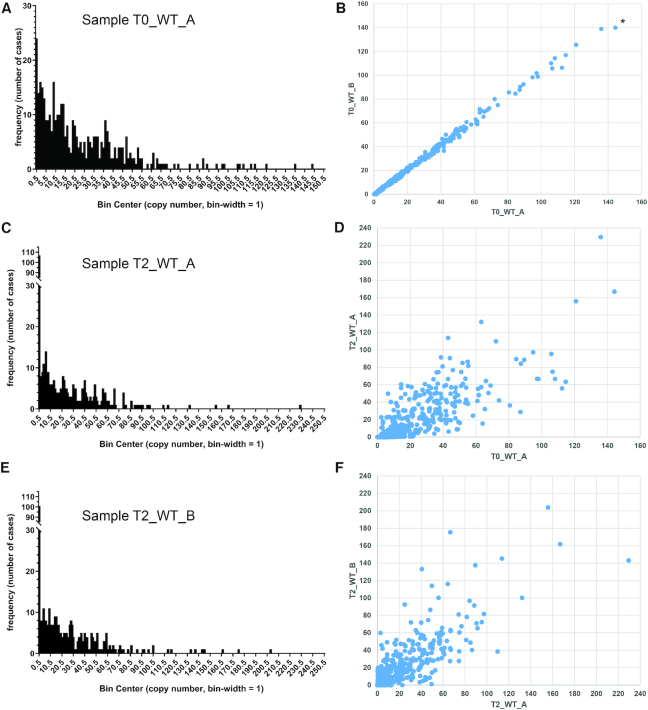
Comparison of average minicircle copy numbers between samples. (**A**) Average copy number of minicircle classes per network for sample T0_WT_A (frequency distribution). Minicircle copy numbers were estimated as follows. First, for preparations of total DNA (see Table 1) the average read depths for core chromosomal regions (i.e. excluding the subtelomeric regions; copy number = 2) and for the maxicircle were determined. From these numbers the average copy number for the maxicircle per cell and kDNA network was determined to be approximately 30. This number was then used to estimate minicircle copy number per network by comparing average read depths for maxicircle and each minicircle class. The numbers on the x-axis indicate the centre of each bin, e.g. 0.5 for 0–0.99 copies per network. In this sample, 24 of the 391 minicircle classes were present, on average, in 0–0.99 copies per network. (**B**) Copy number comparison between samples T0_WT_A and T0_WT_B. Numbers on both axes on the graphs indicate average copy number. Minicircle classes were ordered along the x-axis according to increasing copy number in sample T0_WT_A. For example, the minicircle class indicated by an asterisk (mO_242) had the highest average copy number in sample T0_WT_A (144.3). It also had the highest average copy number in sample T0_WT_B (140.0). (**C**) Average copy number of minicircle classes per network for sample T2_WT_A (frequency distribution). (**D**) Copy number comparison between samples T0_WT_A and T2_WT_A. (**E**) Average copy number of minicircle classes per network for sample T2_WT_B (frequency distribution). (**F**) Copy number comparison between samples T2_WT_A and T2_WT_B.

To calculate the average number of maxicircles and minicircles per network we used coverage (i.e. read depth per kb) of the core regions of the diploid nuclear chromosomes in sample T0_WT_A, which had been prepared from isolations of total cellular DNA (Table 1), as a standard. Average coverage was 46 for core chromosomal regions (2 copies per cell), and 700 for the maxicircle, suggesting an average maxicircle number of ∼30 per network and cell, which agrees well with previous estimates of 20–50 copies per network (6,21). Comparing average read depth per kb for the maxicircle and the 391 minicircles then allowed us to estimate average copy number for each minicircle per network (Figure 1A; Supplementary Table S2). For sample T0_WT_A, average copy numbers within the population ranged from less than 1 copy per network for 24 minicircles (e.g. minicircle mO_068) to 144.3 copies per network for minicircle mO_242. Thus, not all cells in this population had the entire set of 391 minicircles, and copy numbers for minicircles within this population and, presumably, within each network, varied substantially. Similar conclusions had been drawn from earlier, more limited surveys of relative minicircle frequency in *Leishmania* (39,53).

We next estimated the size of the kDNA network by adding up average copy numbers for all minicircles (Supplementary Table S2). These calculations resulted in an estimated average of ∼9,500 minicircles per network and cell for sample T0_WT_A (Supplementary Table S2). We had isolated the DNA samples from asynchronously growing cultures of parasites, which, according to recent models of the *T. brucei* cell cycle, contain only ∼30% of cells with unreplicated kDNA and nucleus (1K1N cells), while ∼25% and ∼45% of cells have partially and completely replicated kDNA genomes, respectively (54). Thus, an unreplicated kDNA network should contain ∼6000 minicircles. These estimates agree well with the earlier estimates of 5000–10 000 minicircles per network that had been based on reassociation kinetics and restriction mapping (6,20,21).

### Minicircles vary substantially in copy number within the network and between cell lines

We next compared estimated minicircle copy numbers between the eight samples listed in Table 1 to assess potential variations caused by clonal selection after transfection and by *in vitro* culturing for extended periods of time (as some samples had used enriched kDNA, coverage of nuclear DNA was not available as a standard in all cases, and we have assumed 30 maxicircles per network for these comparisons; Supplementary Table S2). Supplementary Figure S1 gives an overview of the culturing history and timeline for the eight samples.

The average copy number for each minicircle was similar between the two samples taken at time point T_0_ for cell lines ‘WT’ and ‘L262P’ (Figure 1B; Supplementary Figure S4A; Table S2), confirming that our pipeline of DNA isolation, library preparation, sequencing and mapping preserved relative copy numbers. After 2 weeks of passaging *in vitro* (time point T_1_, ∼40 generations), copy numbers for ‘WT’ had changed noticeably compared to the starting culture, but overall were still well correlated (Supplementary Figure S4B). After an additional 12 weeks of continuous *in vitro* passaging of two parallel cultures, corresponding to an additional ∼250 generations (time point T_2_, samples T2_WT_A and T2_WT_B), we observed an increase in the frequency of minicircle classes with less than 1 copy on average within the parasite population to >100 (Figure 1C, E). The number of cases where a minicircle class could not be detected at all increased from none in T0_WT_A to 41 and 64 in T2_WT_A and T2_WT_B, respectively (Supplementary Table S2). This was despite the fact that overall coverage for kDNA for these samples, and therefore sensitivity of detection of rare classes, was at least 7 times higher than for the starting culture (Table 1). This suggests that culturing of bloodstream form *T. brucei* results in a loss of complexity of the minicircle networks, an important observation. In general, average minicircle copy numbers had diverged substantially for the T_2_ samples, both in comparison to the T_0_ samples (Figure 1D) as well as in comparison to each other (Figure 1F). Similarly, copy numbers for samples representing the two starting cultures at T_0_, T0_WT and T0_L262P, which share the same parental cell line but were derived from distinct transfection experiments and clonal selection regimes (Supplementary Figure S1), are poorly correlated (Supplementary Figure S4C).

Overall, these results confirm suggestions from earlier observations (39,55) and mathematical modelling studies (39,56) that minicircle replication and segregation are not fully deterministic, but instead have a random component which results in plasticity of copy numbers and, over time, in a loss of non-essential minicircle classes and gRNAs from a population.

### Identification of canonical gRNA genes

Next, we passed assembled minicircles through a bespoke pipeline to predict genes encoding ‘canonical’ gRNAs. For the purpose of this study, we defined canonical gRNAs as those that can produce known edited mRNA sequences. Thus, candidate gRNAs can be identified by alignment to those edited sequences, allowing for G-U base pairs (57). To account for potential strain-specific differences, we corrected the published edited sequences in two steps. First, we corrected A, C and G residues based on a maxicircle sequence generated from the same EATRO 1125 kDNA reads used for the minicircle assembly (GenBank submission MK584625). Secondly, we carefully inspected alignments of Illumina reads available from an EATRO 1125 whole-cell RNA sequencing project (45) to these edited sequences to identify potential differences from the published editing patterns. These transcriptome data had incomplete coverage of the mitochondrial mRNAs, but they still offered some guidance. Of note, we identified two alternative versions for A6 and ND8 mRNAs (Supplementary Figure S5, and see below). We then used an alignment-based method to identify matches between minicircle sequences and edited sequence. Note that the precise parameters that govern productive gRNA-mRNA interactions have not yet been defined. For example, it is not known if mismatches or gaps in gRNA–mRNA alignments are tolerated by the editing machinery, although the ‘progressive realignment model’ (13) would predict that they must be tolerated to a certain extent. We therefore used two different sets of rules, both accepting a limited number of mismatches but only one allowing gaps (see Materials and Methods for details). Also, by necessity, the edited mRNA sequences that we used for alignments were an incomplete representation of all fully edited sequences present in strain EATRO 1125. As mentioned above, products of alternative editing continue to be discovered, although the functional significance of these mRNAs and their degree of conservation between different strains of *T. brucei* remain uncertain (reviewed in (12)). Thus, definition of the functional, edited transcriptome is a work in progress. Nonetheless, as shown in Table 2, we identified candidate gRNA genes that provide nearly complete coverage—or, if we allow for some gaps in gRNA/mRNA alignments, complete coverage—for this preliminary set of edited mRNAs, which validates our approach.

**Table 2.** Coverage statistics^a^ for gRNAs predicted by alignment (not allowing gaps / allowing gaps / high confidence set^b^) to minicircle sequences

mRNA^c^	Total gRNAs	Initiator gRNAs	Missing gRNAs	Insertions	Insertions covered	Insertions covered (%)	Deletions	Deletions covered	Deletions covered (%)
A6 v1	130/139/122	1/2/1	0/0/0	443	443/443/443	100/100/100	28	28/28/28	100/100/100
A6 v2	130/137/122	1/1/1	0/0/0	444	444/444/444	100/100/100	28	28/28/28	100/100/100
COX3	227/271/214	1/4/1	0/0/0	547	547/547/547	100/100/100	41	41/41/41	100/100/100
CR3^d^	52/58/49	8/9/8	0/0/0	145	145/145/145	100/100/100	10	10/10/10	100/100/100
CR4^d^	73/81/63	1/3/1	0/0/0	328	328/328/328	100/100/100	45	45/45/45	100/100/100
CYB	6/7/2	2/2/1	0/0/0	34	34/34/34	100/100/100	0	n/a^g^	n/a
MURF2^e^	1/3/1	0/1/0	1/0/1	26	24/26/24	92.3/100/92.3	4	4/4/4	100/100/100
ND3	37/40/35	6/6/6	1/0/1	210	202/210/202	96.2/100/96.2	13	13/13/13	100/100/100
ND7	193/219/189	2/4/2	0/0/0	551	551/551/551	100/100/100	87	87/87/87	100/100//100
ND8 v1	88/113/85	1/4/1	2/0/2	261	249/261/249	95.4/100/95.4	46	46/46/46	100/100/100
ND8 v2	91/112//88	1/4/1	1/0/1	260	258/260/258	99.2/100/99.2	47	47/47/47	100/100/100
ND9	77/85/75	1/2/1	0/0/0	346	346/346/346	100/100/100	21	21/21/21	100/100/100
RPS12	60/78/56	1/4/1	0/0/0	131	131/131/131	100/100/100	28	28/28/28	100/100/100
**total^f^**	**948/1098/896**	**25/42/24**	**4/0/4**	**3026**	**3004/3026/3004**	**99.3/100/99.3**	**324**	**324/324/324**	**100/100/100**

Notes:

^a^Note that editing sites only matched by an anchor sequence are not considered as being covered (for this purpose we considered the first 6 nt of complimentary gRNA sequence as ‘anchor’).

^b^See below for discussion and definition of the high confidence set.

^c^The mRNA encoding COX2 is not included here as its single gRNA is present *in cis* at its 3′ end.

^d^CR3 and CR4 were suggested by Duarte and Tomas (2014) to represent subunits ND4L and ND6, respectively, of respiratory complex I.

^e^Numbers for MURF2 include the maxicircle-encoded gRNA.

^f^The total numbers given take into account editing events and gRNAs that are shared between the two versions of A6 and ND8.

^g^n/a = not applicable.

Not allowing any gaps in the alignments we identified 948 putative minicircle-encoded gRNAs. Together with a maxicircle-encoded MURF2 gRNA, these cover 99.3% of insertions (3004 out of 3026) and 100% of deletions (324 in total). Allowing a limited number of gaps in each alignment, we identified 1098 putative gRNA genes, covering all insertions and deletions. Note that the first 6 bp of the anchor sequence in a gRNA-mRNA pair were not counted towards coverage to provide for the necessary overlap. All alignments are available through JBrowse, a web-based genome browser (58), at http://hank.bio.ed.ac.uk, and for download as plain text files from Figshare (https://doi.org/10.6084/m9.figshare.7756808.v1). As an example, screenshots for alignments of gRNAs to the RPS12 mRNA are shown in Supplementary Figures S6 and S7.

### Identification of non-canonical gRNA genes

**Figure 2.**
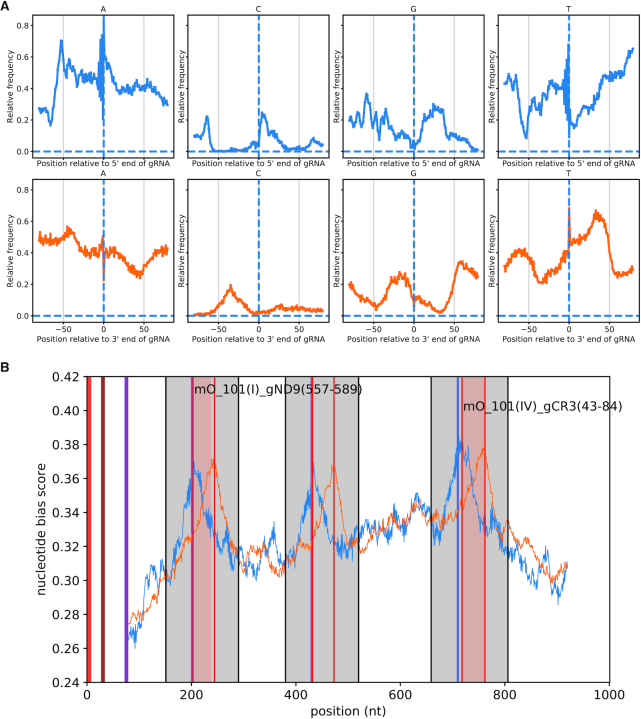
Prediction of non-canonical gRNA genes by nucleotide bias. (**A**) Nucleotide bias of regions upstream and downstream of gRNA genes predicted by alignment to edited mRNAs. Guide RNAs were aligned at their 5′ ends. The frequency of nucleotides at each position from –80 upstream to +80 downstream of the aligned gRNA genes were obtained from the minicircles (top panels). The same procedure was carried out with the gRNA genes aligned at their 3′ ends (bottom panels). An alternative representation of this analysis is shown in Supplementary Figure S8. (**B**) Prediction of a non-canonical gRNA gene on minicircle 101. Grey regions: gRNA gene cassettes identified by nucleotide bias. Orange and blue curves: nucleotide bias scores of 5′ and 3′ aligned gRNAs, respectively, at each position along the minicircle. Pairs of peaks approximately 40 nt apart highlighted by thin, vertical red lines correspond to the predicted 5′ and 3′ ends of gRNAs. Red regions: gRNAs predicted by alignment of minicircles to edited mRNA. Blue vertical lines indicate RYAYA motifs. The first and the third cassette contain predicted canonical gRNAs and the second cassette a predicted non-canonical gRNA. The thick, vertical red, blue and purple lines located within the first 100 nt indicate CSB-1, CSB-2 and CSB-3, respectively.

An analysis of the regions upstream and downstream of the predicted gRNA genes (we only considered gRNAs predicted from non-gapped alignments for this analysis) revealed that these regions were comparatively rich in A/T and poor in G/C (Figure 2A, Supplementary Figure S8). A strong nucleotide bias around gRNA genes has also been reported for *T. cruzi* (59). We next used this nucleotide bias to develop a scoring method that allowed us to predict start and end positions of ‘non-canonical’ gRNA genes that we might have missed due to insufficient complementarity to known edited sequences (see Materials and Methods for details; this method is similar to a method for gRNA prediction that was developed for *T. cruzi* (59) but dispenses with the requirement for a Hidden Markov model). An example is shown in Figure 2B. Using this method, we predicted 367 non-canonical gRNA genes, bringing the total of predicted minicircle-encoded gRNA genes to 1315. Notably, 138 non-canonical gRNAs are classified as canonical if gaps are allowed in the alignment (see above). We have submitted the entire set of 391 minicircles sequences, with all predicted gRNA genes annotated (canonical gRNA genes predicted by non-gapped alignments only), to GenBank, accession number LBIR00000000. A more comprehensively annotated set, not constrained by GenBank formatting restrictions, is available from Figshare (https://doi.org/10.6084/m9.figshare.7756808.v1).

### Organisation of gRNA genes

**Figure 3.**
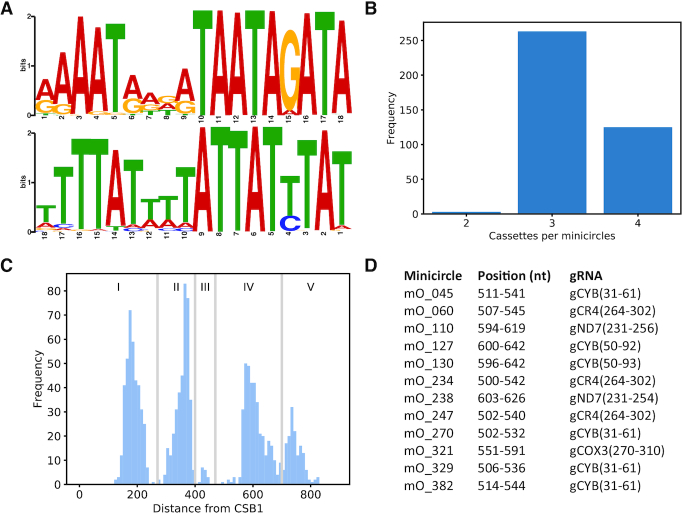
(**A**) Sequence logos for forward (top panel) and reverse (bottom panel) repeats from all 1295 gRNA cassettes. Note that the reverse repeat is shown in 3′ to 5′ direction for ease of comparison with the forward repeat. (**B**) Number of gRNA cassettes per minicircle. (**C**) Cassettes fall into four major categories and one minor category, based on their distance from the CSB-1 motif. **(D)** Minicircles with ‘orphan’ gRNAs, i.e. gRNA genes not flanked by 18-bp inverted repeats. Location in the minicircle and predicted identity of the 12 orphan gRNAs are indicated.

Almost all predicted gRNA genes (1295 out of 1307) are located between pairs of 18-bp inverted repeats that have been reported before (9,22,43) and thus delineate gRNA gene cassettes (Figure 3A). Although the repeat motifs are only semi-conserved, they show a striking symmetry: the positions that are most highly conserved in the forward and reverse repeats, respectively, correspond to each other. This perhaps suggests that these nucleotides are of particular functional importance, for example for recognition by a dimeric DNA binding protein that could be involved in gRNA expression or minicircle recombination. Alternatively, the inverted repeats might facilitate base-pairing interactions between sense and antisense gRNA precursors that could play a role in gRNA maturation (60). Roughly two thirds of minicircles contain three cassettes, and roughly one third contain four cassettes (Figure 3B). Plotting the distance for all cassettes from the CSB-1 motif reveals four predominant regions (Figure 3C, regions I, II, IV and V) and one very minor region (III) where cassettes are located within a minicircle. None of the 391 minicircles had a gRNA cassette in all five regions. Surprisingly, eight canonical gRNA genes were predicted on the antisense (i.e. template) strand of minicircles: mO_030(V)_gND8_v2(496–522), mO_204(I)_gCOX3(213–256), mO_235(I)_gCOX3(203–250), mO_361(I)_gCOX3(210–255), mO_362(I)_gCOX3(211–256), mO_364(I)_gRPS12(107–131), mO_379(I)_gCOX3(215–255) and mO_380(I)_gND9(273–300). It was notable that, with one exception, all were located in the first cassette on their minicircle and five are predicted to match similar regions of the edited COX3 mRNA.

The alignment-based method for gRNA identification also predicted 12 ‘orphan’ gRNA genes not flanked by inverted repeats, all of which are located in the middle region of a minicircle (Figure 3D); 6 of these correspond to gRNAs for apocytochrome b (CYB).

### Relatedness among minicircles

By definition, any two of the 391 minicircles show less than 95% identity overall (not counting 100 bp of the conserved region). Supplementary Figure S9 shows relatedness as a heatmap based on HSP scores from an ‘all vs. all’ BLAST analysis, and Supplementary Table S3 lists the 50 most related minicircle pairs identified in that analysis. Only 16 pairs show more than 80% overall identity (according to EMBOSS Matcher alignments (61)), and the vast majority show limited sequence conservation outside the conserved region and the 18-bp repeats. Nonetheless, some minicircles are clearly related to each other, perhaps suggestive of evolution by sequence drift. For example, the most closely related pair, mO_108/mO_259, are 94.7% identical overall and share nearly identical sets of gRNAs (Supplementary Figure S10). Interestingly, the region in mO_108 corresponding to gCR4(137–182) in mO_259 is annotated as a non-canonical gRNA because a gap interrupts the alignment (indicated by a red arrow in Supplementary Figure S10). Both minicircles have deep coverage with unambiguous reads (see Supplementary Table S1), which rules out sequencing errors as a cause of the discrepancy. This case exemplifies the considerable number of non-canonical gRNAs mentioned above that are reclassified as canonical if gapped alignments are permitted.

### Refined minicircle annotation and gRNA identification by sequencing short mitochondrial transcripts

To assist minicircle annotation and to corroborate identification of the gRNA genes that we had predicted computationally, we next isolated short mitochondrial transcripts (<200 nt) from crude mitochondrial preparations from the same *T. brucei* EATRO 1125 AnTat1.1 cell line used for most of the kDNA preparations (see Table 1). We sequenced three short RNA samples, one from BSF cells, one from PCF cells (obtained by differentiation of the former), and one where RNA samples from BSF and PCF cells had been mixed inadvertently (Table 3). Note that our protocol for cloning small RNAs did not distinguish between transcripts with 5′ monophosphates or 5′ triphosphates, and reads can therefore reflect both primary as well as processed 5′ ends.

**Table 3.** Short RNA samples used in this work

Sample	Total number of read pairs	Merged and trimmed	Mapped to nuclear genome (%)	Mapped to maxicircle (%)	Mapped to minicircles (%)
BSF+PCF, replicate 1	148 935 513	143 870 651	117 351 028 (81.6)	341 908 (0.24)	3 476 778 (2.42)
BSF, replicate 2	79 132 567	52 260 258	41 163 148 (78.8)	122 246 (0.26)	3 008 890 (5.76)
PCF, replicate 2	85 827 714	40 535 598	31 920 884 (78.7)	164 732 (0.33)	490 364 (1.21)

Notes: PCF cells had been obtained by differentiation of an aliquot of the same *T. brucei* EATRO 1125 BSF culture that had been used for preparing RNA for that life cycle stage. Total RNA was isolated from crude mitochondrial fractions, short RNAs (<200 nt) were enriched using the PureLink miRNA isolation kit (ThermoFisher) and barcoded libraries prepared using the Illumina Small RNA kit. Although a single read was expected to cover an entire gRNA sequence, paired 125-bp reads were generated as they offered the opportunity to produce merged reads of improved accuracy. Inadvertently, BSF and PCF libraries for technical replicate 1 had been prepared using adapters with the same barcode and sequenced in the same lane and could therefore not be distinguished (both replicates used RNA isolated from the same crude mitochondrial fraction, but size selection, library preparation and sequencing were carried out separately). Reads for contaminating nuclear RNA were removed by mapping with relaxed stringency to the *T. brucei* TREU927 reference genome (34), version 5.2 (downloaded from www.TriTrypDB.org). Remaining reads were then mapped with high stringency to a *T. brucei* EATRO 1125 maxicircle sequence (assembled as part of this project) and to the 391 assembled minicircles. See Materials and Methods for more technical details.

We carried out four separate mappings to the 391 minicircles: individual mappings with the three read sets listed in Table 3, and one mapping with combined BSF and PCF reads from replicate 2 to serve as a second technical replicate for a combined read set. Plotting read coverage per cassette for these two replicates suggested very good reproducibility, i.e. size selection and library preparation preserved the relative abundance of transcripts very well (Supplementary Figure S11). In general, we observed major peaks of coverage with sense transcripts that correlated very well with the predicted gRNA genes, with much lower coverage in between genes; Supplementary Figure S12 shows minicircle mO_101 as an example (as in Figure 2B). Consistent with earlier reports (60,62) we observed a large degree of heterogeneity in the sizes of gRNAs mapping to a given gene, especially at the 3′ end, and abundant antisense transcripts.

**Figure 4.**
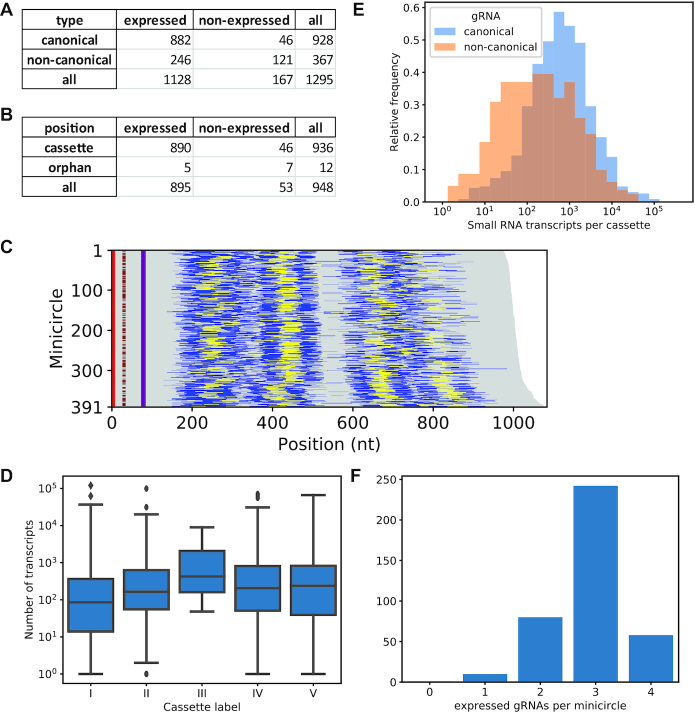
Minicircle expression analysis. (**A**) Expression analysis of minicircle cassettes (defined by nucleotide bias and 18-bp repeats). (**B**) Expression analysis for canonical gRNA genes. Note that the discrepancy between the number of cassettes with expressed putative canonical gRNAs (882) and the number of expressed canonical gRNAs located in cassettes (890) arises because seven cassettes (mO_067 II, mO_164 II, mO_172 I, mO_191 I, mO_232 I, mO_243 I, mO_339 IV) encode canonical gRNAs that are potentially bifunctional, i.e. they match two distinct target mRNA sequences, and one cassette (mO_030 I) appears to encode an additional gRNA in antisense orientation. The 12 ‘orphan’ gRNA genes are located outside cassettes. (**C**) Guide RNA cassette position for all 391 minicircles, sorted according to size. Yellow indicates cassettes confirmed as expressed. CSB-1, CSB-2 and CSB-3 are indicated in red, brown and purple, respectively. (**D**) Distribution of transcript levels for the five distinct cassette locations (I–V; compare Figure 4C) within minicircles. The boxplots show the three quartile values of the distribution along with extreme values. The whiskers extend to points that lie within 1.5 interquartile ranges of the lower and upper quartile, and then observations that fall outside this range are displayed independently. There were no significant differences between positions. (**E**) Distributions of transcript abundances of canonical (blue) and non-canonical (orange) gRNAs (shown for replicate 2, BSF and PCF combined). Canonical gRNAs have just over four times as many transcripts on average than non-canonical gRNAs. (**F**) Numbers of gRNAs expressed per minicircle.

Next, we used the short transcript coverage analysis to identify a subset of ‘high confidence’ (HC) gRNAs, defined as a predicted canonical (based on non-gapped alignments) or non-canonical gRNA that is expressed above a certain threshold in replicate 1 and in at least one of the replicate 2 samples (BSF and/or PCF). We subjectively chose a threshold of a read depth of 0.025% of the total read depth for a given minicircle, thus taking into account a certain level of ‘background noise’ observed for most minicircles as well as the vast differences in average copy number for minicircles (N.B.: a caveat of this approach is that the underlying assumption that functionality of a gRNA requires steady-state levels above a certain threshold could be wrong, as it has been suggested that gRNAs might get consumed in the editing process (8); hence, low abundance of a gRNA could conceivably be a consequence of rapid use and turnover during editing). Minicircle mO_101 again serves as an example to illustrate the data (Supplementary Figure S13); the coverage analysis for sense and antisense transcripts for all minicircles and data sets is available on Figshare (https://doi.org/10.6084/m9.figshare.7756808.v1); Supplementary Table S4 lists the expression data for all gRNAs and transcriptome datasets as sortable barcodes. According to these criteria, of the 1,295 cassettes, 1,128 are expressed (Figure 4A, C), including five that are predicted to encode a canonical gRNA in antisense orientation: mO_030(V)_gND8_v2(496–522), mO_204(I)_gCOX3(213–256), mO_235(I)_gCOX3(203–250), mO_361(I)_gCOX3(210–255) and mO_379(I)_gCOX3(215–255). Position of a cassette on a minicircle does not appear to significantly influence expression levels (Figure 4D). Interestingly, while, according to the cut-off applied, 95% of cassettes predicted to encode a canonical gRNA were expressed (882 out of 928), this was the case for only 67% of cassettes predicted to encode a non-canonical gRNA (246 out of 367), consistent with generally lower expression levels for non-canonical gRNAs (Figure 4E).

As observed previously (9), within cassettes the distance of the forward 18-bp repeat to the start of the gRNA gene is remarkably conserved, potentially reflecting a role of the repeats in transcription initiation: for more than 94% of gRNA transcripts in our study (regardless of whether canonical or non-canonical), the distance between the 3′ end of that repeat and the 5′ end of the transcript was 30–32 bp (Supplementary Figure S14, left panels).

The distribution of the number of gRNAs expressed per minicircle was slightly shifted to the left compared to the distribution of the number of cassettes per minicircle (Figure 4F, compare Figure 3B), reflecting the fact that not all cassettes give rise to transcript levels above the chosen cut-off. Most minicircles expressed three gRNAs (Figure 4F). Of the 948 putative, minicircle-encoded canonical gRNAs that we identified, 12 were ‘orphans’, i.e. were located outside cassettes (Figure 4B, see also Figure 3D). Only five orphan gRNAs reached transcript levels above the cut-off for their respective minicircle (mO_060_gCR4(264–302), mO_110_gND7(231–256), mO_130_gCYB(50–93), mO_238_gND7(231–254), and mO_329_gCYB(31–61); Supplementary Table S4). Thus, the complete set of HC gRNAs that we identified comprised 895 canonical and 246 non-canonical gRNAs. The canonical HC gRNAs form the basis for the following coverage analysis of edited mRNAs.

### Coverage of known edited sequences

**Figure 5.**
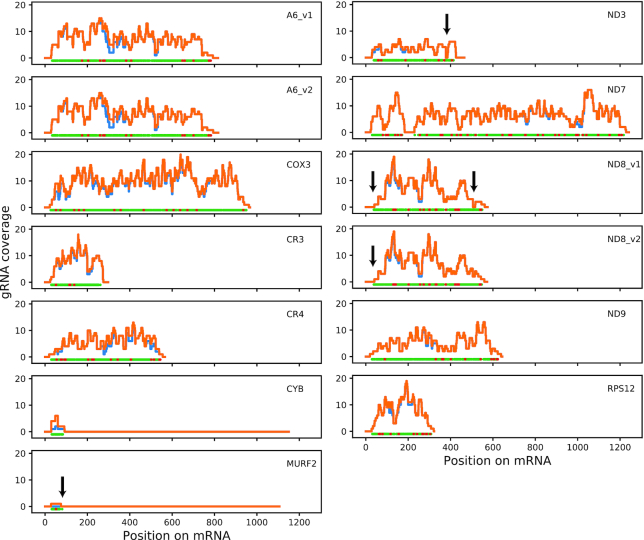
Redundancy of editing site coverage. For each position on each edited mRNA we counted (i) the number of gRNAs found by minicircle alignment that cover that position (orange line), and (ii) the subset of HC gRNAs that cover that position (blue line). At the bottom of each panel, green dots represent U-insertion site and red dots represent U-deletion sites. Arrows indicate gaps of coverage (gRNA coverage = 0) within editing domains.

An overview of the number of editing sites covered by the HC gRNA set is shown in Table 2. The vast majority of computationally predicted gRNA genes (94.4%) produced transcript levels above the threshold. All deletion sites and 99.3% of insertion sites are covered by at least one gRNA, and initiator gRNAs were identified for all mRNAs except MURF2. Interestingly, we often found only a single initiator gRNA for a given mRNA species, despite the fact that we observed substantial redundancy for subsequent (i.e. upstream) editing sites (Figure 5). Notable exceptions with multiple initiator gRNAs were CR3, ND3 and ND7. To cover all editing sites we estimate that at least four additional gRNAs would be necessary (Table 2, Figure 5).

### Respiratory complex I / NADH:ubiquinone oxidoreductase

In total, at least eight subunits of this complex are encoded in kDNA, and four of these require pan-editing for maturation. In addition, C-rich open reading frames CR3 and CR4 have been proposed to encode subunits ND4L and ND6 of this complex (63), but as this identification is tentative these genes are discussed below under ‘*Unidentified open reading frames*’. The HC set includes gRNAs for complete editing of ND7 and ND9 only, with substantial redundancy for most editing sites. A complete set of gRNAs for these mRNAs was also reported by Koslowsky et al. (25), although with much less redundancy and with an interesting difference: editing of the 5′-most editing site of the ND7 3′ editing domain (position 231 in the fully edited mRNA) in the previous study was predicted to be directed by a long gRNA that also directed editing of the preceding editing sites, which are separated from that last editing site of the domain by 16 nt of never-edited sequence. The only gRNAs identified in our HC set that are capable of directing editing of that 5′-most site are two relatively highly expressed orphan gRNAs, mO_110(Orphan)_gND7(231–256) and mO_238(Orphan)_gND7(231-254) (see also Figure 3D; minicircles mO_110 and mO_238 are clearly related as they encode sets of gRNAs that direct editing of equivalent regions of mRNAs, although overall sequence identity is <70%).

For ND3, we identified six potential initiator gRNAs, but no gRNAs that would bridge the gap to the next set of gRNAs (unless we allowed for gaps in the alignments), leaving 6 U insertion sites uncovered (Figure 5). That region of edited ND3 has been suggested to be variable, with no readily identifiable consensus (64). Our inspection of whole genome RNA-seq data for EATRO 1125 (45) also failed to identify any obvious alternative sequences. We conclude that the fully edited ND3 3′ sequence is either highly heterogeneous, or if a consensus sequence exists it remains to be discovered.

For ND8, our inspection of the EATRO 1125 transcriptome suggested two potential variations from the reported consensus (65), here referred to as ND8_v1 and ND8_v2 (Supplementary Figure S5B and C). The coding sequence of ND8_v1 is identical to the published consensus and has complete gRNA coverage for that region, but both ND8_v1 and ND8_v2 differ from the published sequence by introducing additional single Us at two different sites: just downstream of the published first editing site (nt 550 in ND8_v1, Supplemental Figure S5C) and between G534 and A535 in the published sequence; the latter variation has also been reported for strain Lister 427 (14). We found an initiator gRNA for this alternative sequence (mO_004(V)_gND8(519–562)), but not for the published sequence. ND8_v1 and ND8_v2 differ in two ways: (i) the EATRO 1125 transcriptome data suggested a polymorphism in the edited sequence at nt 379, with one population of transcripts corresponding to the published sequence (here represented by ND8_v1) and another population that is characterised by deletion of an additional U (represented by ND8_v2) (Supplemental Figure S5B). The change in reading frame in ND8_v2 would result in an alternative protein product that is 27 amino acids shorter and differs from the published sequence in its final 14 amino acids. We identified putative gRNAs for both variants. (ii) The EATRO 1125 transcriptome suggested an edited ND8 mRNA that differs from the published sequence in three editing sites just 3′ to the stop codon (nt 505–515, Supplemental Figure S5C). We identified gRNAs for this alternative sequence (here again represented by ND8_v2), but not for the published sequence. In summary, we found evidence that in EATRO 1125 there are at least two versions of edited ND8 mRNA that would result in two different protein products. Although we report an editing pattern in the 3′ untranslated region that differs from the original published sequence, it shows some similarities to later studies.

### Respiratory complex III / cytochrome *bc*_1_ complex

Only a single subunit of this complex, apocytochrome *b* (CYB), is kDNA-encoded. The mRNA requires editing by insertion of 34 Us into its 5′ region for maturation. We identified six putative gRNAs (non-gapped alignments), all of which are ‘orphans’ (Figure 3D). Two gRNAs met criteria for the HC set (mO_130(Orphan)_gCYB(50–93) and mO_329(Orphan)_gCYB (31–61)) and overlap sufficiently to account for all editing events (Table 2). It had previously been reported that the initiating gRNA for CYB is not flanked by 18-bp repeats (22,66), and we now show that this appears to be the case for all CYB gRNAs encoded in this strain's kDNA.

### Respiratory complex IV / cytochrome *c* oxidase

Three subunits of this complex are kDNA encoded, and two require RNA editing for maturation. Subunit 2 (COX2) is unique as its limited editing of four insertion sites is guided by a region within the 3′ UTR of the mRNA, i.e. the gRNA is encoded *in cis* on the maxicircle. Subunit 3 (COX3) requires extensive editing for maturation (Table 2). We identified 214 putative gRNAs in our HC set (the highest number for any mRNA) that provide complete coverage, typically with substantial redundancy. Notable exceptions are the most 5′ and 3′ editing sites, which are covered by a single species of ‘terminator’ and initiator gRNA, respectively (Figure 5). However, in general, and in contrast to what was reported for strains EATRO 164 and Lister 427 (14,25), we observed similar transcript abundances for initiator gRNAs compared to extender gRNAs (Supplementary Figure S15).

It has been proposed that an alternative gRNA directs insertion of one fewer U between G431 and A435 (coordinates of the edited sequence), and that this results in production of an alternative protein product, AEP-1 (19,67). The version of our gRNA identification pipeline that permits a limited number of gaps in gRNA-mRNA alignments generates an extended alignment between HC gRNA mO_262(II)_gCOX3(434–473) and the COX3 mRNA that could direct this alternative editing event (compare alignment text files ‘COX3_nogaps_exp’ and ‘COX3_gaps_all’, available on Figshare), but we also identified numerous gRNAs that would direct canonical editing of the same region. Thus, competition between canonical and alternative editing events could potentially generate a mixture of COX3 and AEP-1 proteins.

### Respiratory complex V / F_1_F_O_-ATP synthase

A single subunit of this complex, subunit 6 (A6; or *a*, in mammalian nomenclature), is kDNA-encoded. As mentioned above, we found evidence for two different versions of fully edited A6 in the EATRO 1125 transcriptome, here referred to as A6_v1 and A6_v2; both versions differ from the published A6 sequence (Supplementary Figure S5A). The differences between these mRNA sequences only concern the 3′ UTRs. We identified gRNAs for complete editing of both versions in the HC set. Indeed, alternative use of two different initiator gRNAs (mO_345(II)_gA6_v1(750–793) versus mO_229(II)_gA6_v2(746–794) is sufficient to explain the difference in editing patterns (Supplementary Figure S5A). Minicircles mO_345 and mO_229 otherwise contain identical sets of gRNAs, despite <65% overall sequence identity, suggesting that these two minicircles share a common ancestor, and that subsequent sequence divergence generated two distinct initiator gRNAs, and, consequently, different versions of the edited A6 mRNA. Remarkably, a similar scenario was reported for two other strains of *T. brucei*, EATRO 164 and Lister 427, with the alternative A6 sequence in those studies being identical to our version A6_v2 (14,25). The conservation of this feature in *T. brucei* strains with distinct origins suggests that it might be of functional relevance.

### Mitoribosome

Two protein components of the mitoribosome are kDNA-encoded (68); one subunit, RPS12, undergoes extensive editing. We identified 56 HC gRNAs that cover all editing sites. Again, the only exceptions to the typically highly redundant coverage are the editing sites at the 5′ and 3′ ends of the editing domain. These are each covered by a single species of gRNA (Figure 5).

### Unidentified open reading frames

Three kDNA-encoded open reading frames are of uncertain or unknown function: CR3 and CR4 potentially encode subunits of respiratory complex I (63) (see above); no putative function has been identified for mitochondrial unidentified open reading frame 2 (MURF2). For CR3 and CR4, which are short but require extensive editing, complete complements of gRNAs are included in our HC set. CR3 is unusual insofar as it has eight putative initiator gRNAs, the highest number among all mRNAs (Figure 5). CR3 is also unusual among pan-edited mRNAs in that it has no editing sites within its 3′ UTR. Note that for CR4 we used the edited sequence reported for the BSF stage of the parasite; editing of the 5′ region of the mRNA in PCF parasites may be different or incomplete (69).

Editing of MURF2 is limited to a relatively short region near the 5′ end of the mRNA and presumably directed by two gRNAs. The second (and terminal) gRNA is encoded by a distinct gene on the maxicircle—the only such case described for *T. brucei*—and we found a large number of short transcripts that mapped to the expected region between the CR4 and ND4 genes. However, we could only find a candidate for an initiator gRNA, an abundant transcript encoded by cassette II on mO_112, if we allowed gaps in the alignment (alignment text file ‘MURF2_gaps_all’). The match is of poor quality, however, and it is very doubtful if this transcript is indeed the MURF2 initiator gRNA. Other deep sequencing analyses of the gRNA population in *T. brucei* have also failed to identify a good candidate for a MURF2 initiator gRNA (25,26).

### Preliminary comparison of gRNAs detected in BSF and PCF parasites

We compared coverage of editing sites by gRNAs considered as ‘expressed’ in BSF and PCF, respectively, applying the same cut-off criterion based on minicircle-specific read coverage described above (Supplementary Table S4, replicate 2). Note that the PCF parasites had been obtained after differentiation of the BSF cells. We found 107 additional gRNAs in the PCF set (Supplementary Table S4), despite the fact that read depth was ∼6 times higher for the BSF sample (Table 3). This higher number of gRNAs resulted in increased redundancy but did not translate into substantially better coverage: in both sets, coverage for seven mRNAs remained slightly incomplete because 1 and 2 gRNAs for each of these transcripts were only found in one set, but not the other (Supplementary Table S5). Both samples lacked complete coverage for MURF2, ND3 and the two versions of ND8. The BSF sample also did not have complete coverage for CR4, CYB and ND7, while the PCF sample lacked single gRNAs for ND9 and the two versions of A6. It seems likely that these cases of incomplete coverage reflect incomplete sampling of the gRNA population in these single samples rather than evidence of stage-specific regulation. Future studies should include multiple biological replicates for each life cycle stage to investigate potential stage-specific differences in more depth and with sufficient statistical power.

To summarize, our HC gRNA set provides complete coverage for eight of the eleven mRNAs that depend on minicircle-encoded gRNAs for full editing, and for most editing sites we identified multiple gRNAs. These gRNAs also suggest potentially interesting cases of alternative editing events that deserve more in depth investigation in the future. Gaps in the coverage of edited ND3 and ND8 mRNAs are likely a consequence of errors in the consensus sequences used for the alignments. Our preliminary analysis of BSF- and PCF-specific subsets did not provide evidence for a major role of gRNA abundance in stage-specific RNA editing.

### Biogenesis of gRNAs and short antisense transcripts is not symmetrical

**Figure 6.**
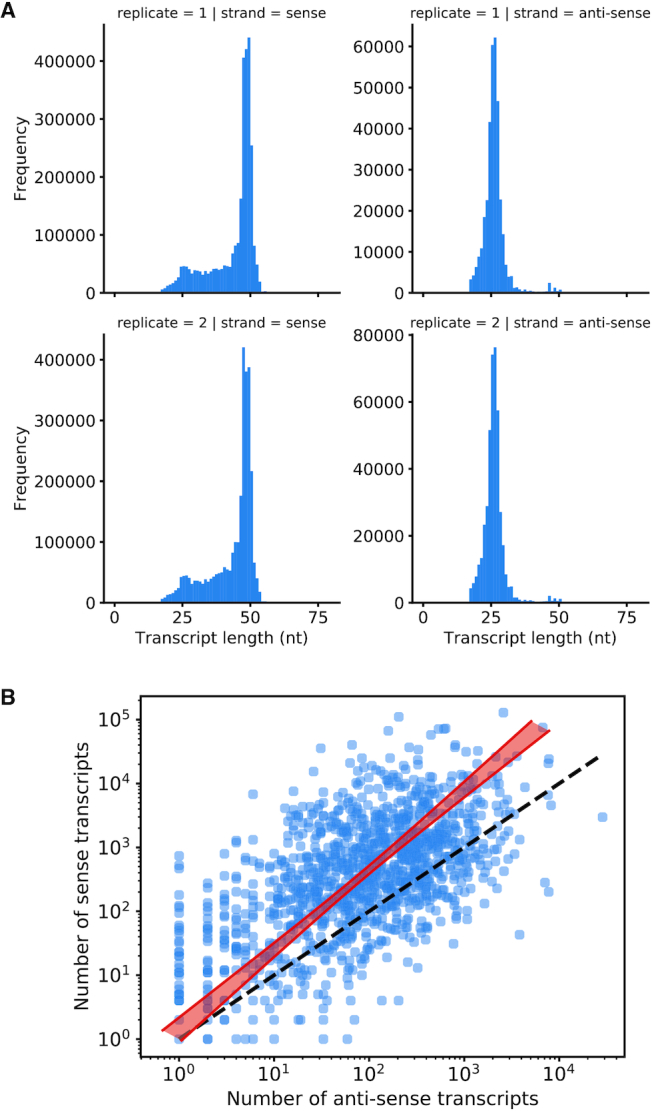
Basic characteristics of short antisense transcripts. (**A**) Length of gRNAs (left panels) and short antisense transcripts (right panels) mapping to canonical and non-canonical HC gRNA genes. For replicate 2, BSF and PCF samples were combined. U-tails had been removed from transcripts for this analysis. (**B**) Transcript abundances are correlated between sense and antisense transcripts of the same gRNA gene. The ratio of the number of sense transcripts to antisense transcripts for a given gRNA gene is about 2 at low abundances but rises to ∼15 at the highest abundances. The gradient of the orthogonal distance regression is significantly different from 1 (*t* = 5.5, *P* < 0.001). All transcripts aligned to gRNA cassettes were used. The dashed black line represents equality of transcript abundance.

Earlier studies had discovered that minicircles also give rise to antisense transcripts (62,70). A model was proposed in which hybridisation of sense and antisense transcripts from the same minicircle forms duplex intermediates that define the boundaries for largely symmetrical processing by a macromolecular complex, termed the mitochondrial 3′ processome (MPsome), to produce mature gRNAs and short antisense transcripts, the latter of which are then degraded (60). As a final step of our analysis we compared some basic characteristics of short sense and antisense minicircle transcripts mapping to HC gRNA genes. While, as reported above, the distribution for distances between the forward 18-bp repeat to the begin of the gRNA gene showed a strong peak at 30–32 bp (Supplementary Figure S14, left panels), the corresponding distances for antisense transcripts to the reverse 18-bp repeat showed a normal distribution that peaked around 40 bp (Supplementary Figure S14, right panels). In addition, while most gRNAs in our dataset had a length of 40–50 nt (not counting U-tails; Figure 6A, left panels), most antisense transcripts were only 20–30 nt long (Figure 6A, right panels). Abundance of gRNA and corresponding short antisense RNA for a given gene showed some correlation but was biased towards gRNAs, rising from a ratio of 2 for low-abundance gRNAs to about 15 for high abundance gRNAs (Figure 6B). Interestingly, there were some striking exceptions to this general rule. For example, gRNA genes mO_097(II)_non-canonical, mO_111(I)_gND9(199–246) and mO_176(II)_non-canonical showed dramatically higher abundance of anti-sense compared to sense transcripts (see file ‘sense and antisense coverage for all 391 mOs.pdf’ in the additional dataset on Figshare).

This analysis suggests that biogenesis of coding-strand encoded gRNAs and the corresponding template-strand encoded antisense transcripts is, at least in part, asymmetrical, i.e. there appear to be distinct differences in how these classes of transcripts are generated.

## DISCUSSION

The kinetoplast (or kDNA) of the single-cellular parasite *T. brucei* is one of the most complex mitochondrial genomes in nature, but previous estimates of its complexity relied on indirect methods or extrapolations (20,21). Its precise content remained unknown. We have used a deep-sequencing approach to generate an essentially complete assembly for a parasite strain that is able to go through the entire life cycle, an important consideration as the different developmental stages in mammal and insect vector require different sets of mitochondrial genes. We have identified 391 distinct minicircle molecules that differ greatly in average copy number, and we show that these account for nearly all minicircles that were present in appreciable numbers in the parasite populations that served as starting material for our project. Some of the low abundance minicircle classes were, on average, present in less than one copy per cell, which demonstrates the efficiency of our assembly. Using computational approaches we refined the description of the structural characteristics of *T. brucei* minicircles and the encoded gRNA genes (detailed below). We identified 1307 putative gRNA genes that not only provide coding capacity for 99–100% of known (i.e. canonical) U insertion and deletion events, but also include 367 candidates for potentially novel (non-canonical) events. A complementary analysis of the small RNA transcriptome in the same cell line allowed us to confirm 1141 of the predicted genes as expressed. Finally, our dataset constitutes a rich resource for future studies that, for example, might aim to explore the dynamics of minicircle evolution and the fidelity of their inheritance, or that seek to reveal the potential significance of gRNAs coding for alternative editing events.

By necessity, our distribution of the assembled minicircle contigs into 391 classes is somewhat arbitrary as there is no universally accepted definition of a minicircle class. We used a cut-off of 95% identity (not counting 100-bp of the conserved region) as criterion to distinguish distinct classes, but it is clear from our study as well as from earlier reports (22,66) that some classes are closely related and contain the same or very similar gRNA genes, in the same order. A detailed phylogenetic analysis of the minicircles identified here, and a comparison with minicircles identified in other studies, was outside the scope of the present study but should be highly rewarding.

Our study confirms the notion of plasticity in minicircle class copy numbers from studies in *Leishmania* (39) and *Crithidia* (71). After only two weeks of passaging *in vitro* (∼40 generations), copy numbers for most minicircle classed had changed considerably. After 12 weeks of *in vitro* culture, the number of minicircle classes with, on average, less than one copy per cell had increased from ∼20 to >100. The precise passaging history of the strain used for our study is unknown, although care has been taken to avoid prolonged culturing as this is known to result in loss of pleomorphism (72). Nonetheless, extrapolation of our findings suggests that stocks of this strain with shorter passaging history since the original isolation should have a minicircle diversity that is greater than the 391 classes determined here. Such rapid loss of minicircles may support a random segregation model for minicircle inheritance to daughter cells (56). In-depth comparative analyses of minicircle diversity from multiple samples that represent different time points in passaging history, combined with mathematical modelling approaches (39,56), should allow to determine if minicircle segregation is purely random and to precisely quantify any deterministic component that may exist. Our observation of rapid minicircle loss is also relevant in the context of the fact that long slender BSF *T. brucei* require only two of the mRNAs that depend on minicircle-encoded gRNAs for expression (A6 and RPS12) (29). Thus, many minicircles are not expected to be under selection while the parasite resides in the mammal and are therefore prone to loss (73–75). This would be expected to be particularly relevant in cases of chronic infection, for example human infection with *T. brucei gambiense* (76). Advances in single cell sequencing technology promise to provide a technology that will be able to reveal heterogeneities in minicircle diversity within parasite populations, which could be an important factor in transmission success and dynamics.

The general structure of minicircles has been known for a while. A conserved region is involved in replication and contains three conserved blocks of sequence, CSB-1, -2 and -3; the latter, GGGGTTGGTGTA, is also known as the universal minicircle sequence (22,42,51). Upstream of the conserved region is a region characterised by periodically repeated A-tracts that causes a bend in the DNA (52) and may aid organisation of minicircles into the kDNA network (3). *T. brucei* minicircles are ∼1 kb in size and typically contain 3–4 gRNA gene ‘cassettes’ on the sense (coding) strand (defined by the orientation of the CSBs), each framed by imperfect 18-bp inverted repeats (9,22,43). Our study confirms these observations on minicircle structure, refines some characteristics and introduces some new features. As expected, CSB-1 was 100% conserved, but three minicircles classes contained a slight variation of CSB-3, GGGGTTGATGTA. To predict gRNA genes for known (i.e. canonical) editing events we aligned minicircle sequences to fully edited mRNAs, allowing for G:U base pairs (22,57). However, as it is unknown to what extent mismatches or gaps are permitted in productive gRNA-mRNA alignments, even canonical gRNAs cannot currently be predicted with certainty (25). We only obtained complete coverage of all canonical editing sites (allowing at least 6 nt overlap between gRNAs for anchor binding) if we permitted a certain number of gaps in the alignments. However, even using stringent alignment conditions without gaps and very few mismatches, more than 99% of insertion editing sites and all deletion editing sites were covered by gRNAs. Minicircles annotated using these stringent conditions were used for subsequent analyses. The vast majority of minicircles indeed contained 3–4 gRNA gene cassettes, framed by 18-bp inverted repeats, although we defined five distinct cassette positions based on their distance from CSB-1. As an additional characteristic of these gRNA cassettes we discovered a distinct nucleotide bias (rich in A/T and poor in G/C) in regions immediately upstream and downstream of the gRNA coding sequences. This is reminiscent of *T. cruzi* gRNA genes, although in that species the upstream and downstream regions are comparatively richer in G (59). We exploited this nucleotide bias to predict 367 putative ‘non-canonical’ gRNA genes that do not match known edited sequence. A surprising finding was the apparent existence of gRNA genes encoded on the bottom (template) strand of eight cassettes. In one case, cassette V of mO_030, two different gRNA genes are predicted for the sense and antisense strand. We also found 12 putative ‘orphan’ gRNA genes, i.e. genes located outside of cassettes, including the CYB gRNA genes reported previously (66).

As a means to validate our gene predictions, we generated complementary small transcriptome data for the same *T. brucei* EATRO 1125 stock, before and after differentiation from the long slender BSF to the PCF, and mapped transcript reads to the 391 minicircles. Obtaining small RNA transcriptomes from these two life cycle stages seemed useful as their energy metabolisms differ substantially and, as a consequence, these developmental forms depend on the expression (and thus editing) of distinct subsets of mitochondrial genes (26,73,77). As mentioned above, BSF *T. brucei* require editing of only two mitochondrial mRNAs for survival (29). Indeed, RNA editing appears to show a developmental pattern between BSF and PCF cells (reviewed in (73)). Although earlier studies had not found a correlation of the editing status of a given mRNA species with the abundance of corresponding gRNAs (66,78), comparative analysis of gRNA transcriptomes of BSF and PCF *T. brucei* strain EATRO 164 had suggested substantial stage-specific differences in gRNA abundance (25,26). Our initial intent had been to generate two replicates for each life cycle stage to permit a more robust comparison, but BSF and PCF samples for one replicate inadvertently got mixed. We therefore focused our analysis primarily on the validation of predicted gRNA genes.

In general, for most minicircles we observed a striking correlation of sharply increased read depth (peaks) with predicted gRNA genes, usually on both the sense (forward, or coding) and the antisense (reverse, or template) strand, although there were exceptions (i.e. lack of reads for predicted genes as well as peaks of read depths in regions with no predicted gene). Using a consistent criterion for expression that considered reproducibility and minicircle-specific read depth, we confirmed 882 canonical gRNA gene cassettes as expressed, including five of the gRNAs that are predicted to be encoded in antisense orientation. We also confirmed expression of five orphan genes. This set of ‘high confidence’ gRNAs covered more than 99% of known U insertion sites and all known U deletion sites, in most cases with considerable redundancy (i.e. most editing sites were covered by multiple gRNAs).

Analysis of characteristics of antisense RNAs in our short transcriptome data revealed inconsistencies with a model that had suggested essentially symmetrical processing of gRNAs and antisense RNAs (60). While initiation of gRNAs occurs at a remarkably conserved distance of 30–32 nt from the forward 18-bp repeat, as reported previously (9), we found that the corresponding distances for antisense transcripts to the reverse 18-bp repeat showed a normal distribution that peaked around 40 nt (a notable exception were the putative gRNAs produced from the antisense strand, these initiate 30–32 nt from the reverse 18-bp repeat). Antisense transcripts were typically ∼20 nt shorter than gRNAs, with shorter U-tails. Overall, these data suggest asymmetries in the biogenesis of gRNAs and the corresponding short antisense transcripts. To what extent these differences reflect transcription vs. processing by the MPsome complex (60) is an interesting question for future studies.

One of the most interesting discoveries in our study was the large number of putative gRNA genes that could not be readily matched to known sequences of edited mRNAs. Our small RNA transcriptome analysis confirmed 246 of these non-canonical gRNA genes as expressed. Like canonical gRNAs, they typically initiate 30–32 bp downstream of the 3′ end of the forward 18-bp repeat (9), have a similar bias in their 5′ initiation sequence, and have 3′ oligo(U) tails of similar length. On the other hand, we found that canonical gRNAs were on average ∼4 times more abundant than non-canonical gRNAs and that a considerably larger proportion of non-canonical gRNA genes could not be validated by the presence of corresponding transcripts. The existence of non-canonical gRNAs raises a number of questions. Are these molecules substrates for the editing process? If so, do they create alternative edited sequences of functional relevance, or are they solely responsible for ‘misediting’ events that have been observed (79–81) and that may or may not get corrected subsequently? The question of alternative editing events of functional significance remains controversial, despite accumulating evidence for the persistence of non-canonical editing patterns in mRNA populations (17,82–84) and evidence that at least one alternatively edited mRNA is translated into a protein, AEP-1 (19,67). We find that some non-canonical gRNAs are closely related to canonical ones, and that less stringent conditions for gRNA identification reclassify many non-canonical gRNAs as canonical. Thus, at least some non-canonical gRNAs may have evolved fairly recently from canonical ones by accumulating mutations that increase the number of mismatches or gaps. Nonetheless, our study provides some support for the existence of the production of functionally relevant mRNAs by alternative editing. One example is evidence that alternative initiator gRNAs for A6 produce two alternative mRNAs in *T. brucei* EATRO 1125. As the exact same alternative sequences have been reported in other strains of *T. brucei* (14,25) it seems reasonable to assume that these events must be of some functional importance, even though the editing events only affect the 3′ UTR of the mRNAs and not the predicted protein sequence. We also identified a gRNA that could direct the alternative editing event reported to result in production of AEP-1, although this scenario would require the tolerance of gaps in alignment between that gRNA and the COX3 mRNA. We only discovered gRNAs for these potential alternative editing events because we knew what to look for. Clearly, one important step towards solving the question of alternative editing will be the production of high quality mitochondrial mRNA transcriptomes for multiple strains of *T. brucei* and other species.

## DATA AVAILABILITY

Additional data are available on Figshare (https://doi.org/10.6084/m9.figshare.7756808.v1) and http://hank.bio.ed.ac.uk. Scripts for bioinformatics analyses are available in the GitHub repository https://github.com/nicksavill/kDNA-annotation.

The *T. brucei* EATRO 1125 AnTat1.1 maxicircle conserved region and the 391 assembled minicircles are deposited in GenBank (accession numbers MK584625 and LBIR00000000, respectively).

DNA and RNA sequencing data are deposited in NCBI SRA under BioProject ID PRJNA530667.

## Supplementary Material

gkz928_Supplemental_Files

## Notes

Present address: Sinclair Cooper, Synpromics Ltd, Roslin Innovation Centre, Easter Bush Campus, Midlothian EH25 9RG, UK.
