# Supplementary material for: Assembly and annotation of the mitochondrial minicircle genome of a differentiation-competent strain of Trypanosoma brucei
Source: Nucleic Acids Res. 2019 Oct 30;47(21):11304–25. doi: 10.1093/nar/gkz928 (PMC6868439; doi:10.1093/nar/gkz928)
Supplement: gkz928_Supplemental_Files [file gkz928_supplemental_files.zip › Supplementary Figures.pdf]

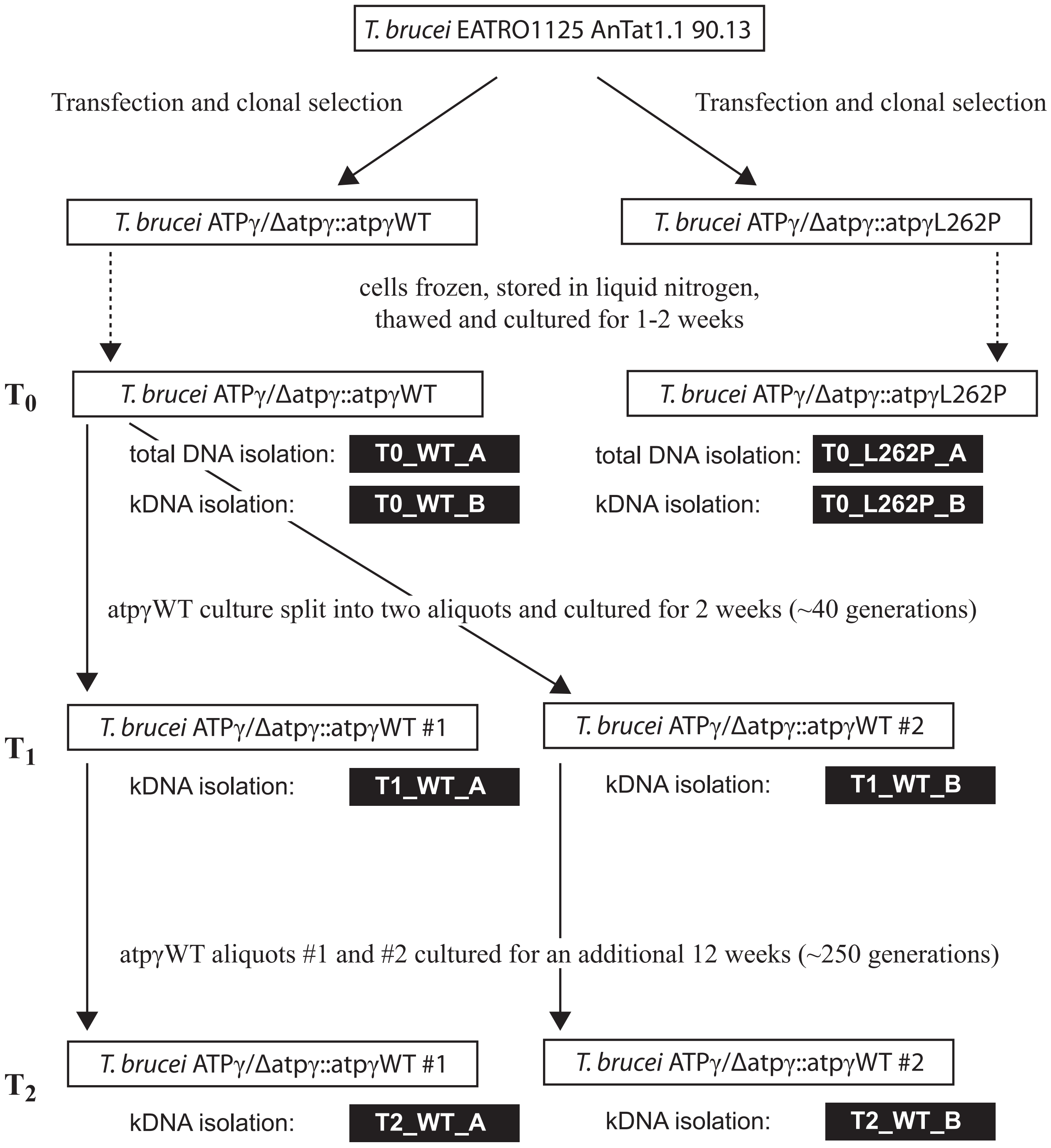

Supplementary Figure S1. Timeline for the eight samples used in this study (compare Table 1). Samples are indicated as black boxes with white text.

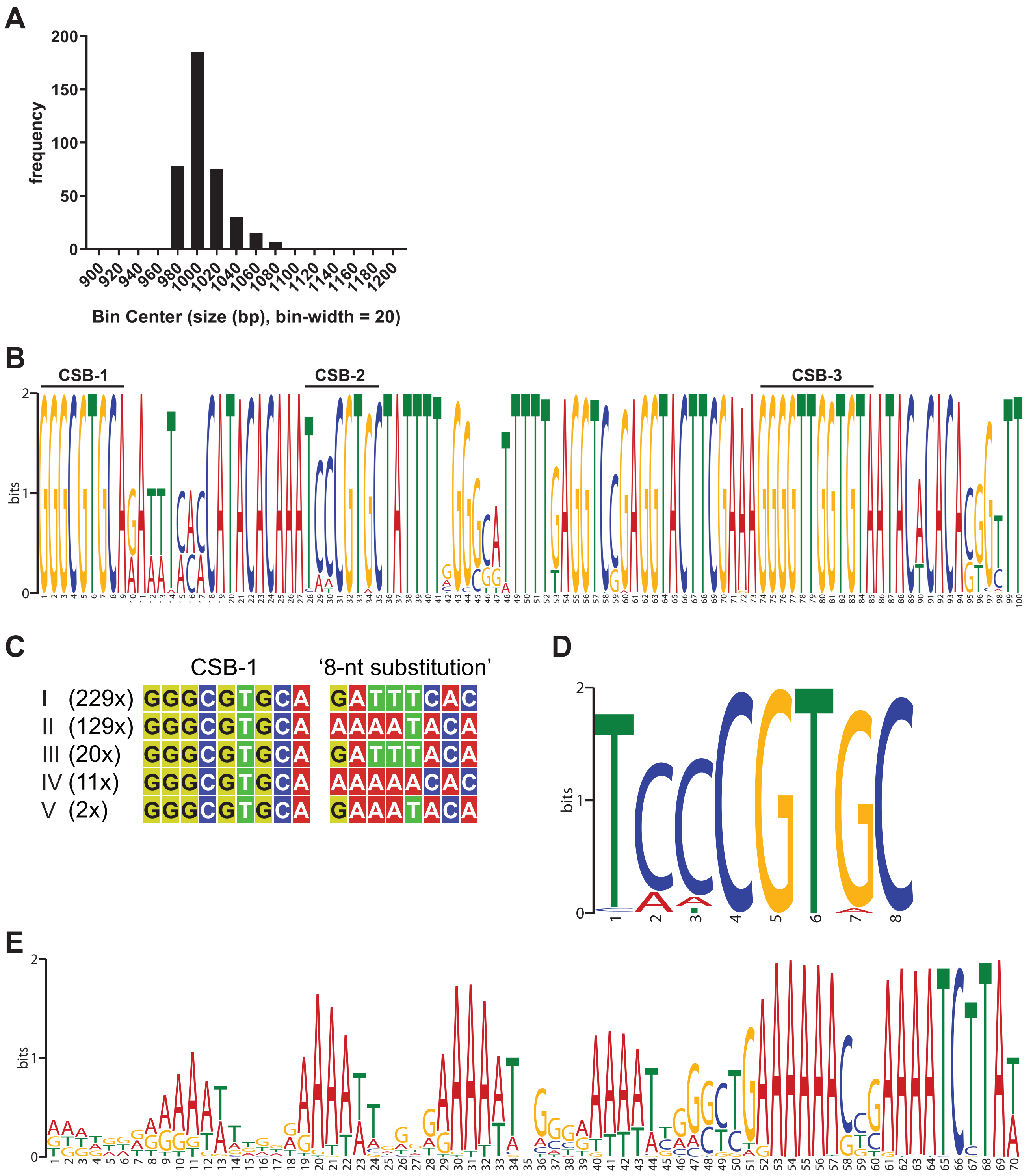

Supplementary Figure S2. **(A)** Size distribution of the 391 assembled minicircles. Minicircles were distributed into 'bins' of 20-bp increments. The numbers on the x-axis indicate the centre of each bin. **(B)** Sequence logo for the conserved region, starting with conserved sequence block CSB-1. CSB-2 and CSB-3 are also indicated. **(C)** The 391 minicircles fall into two major and three minor groups, based on the 8-bp sequence immediately downstream of CSB-1. **(D)** Sequence logo for the CSB-2 motif, generated with the MEME Suite (Bailey et al., 2009). **(E)** Sequence logo for the region of A-tracts immediately upstream of CSB-1. Logos were generated with the MEME Suite.

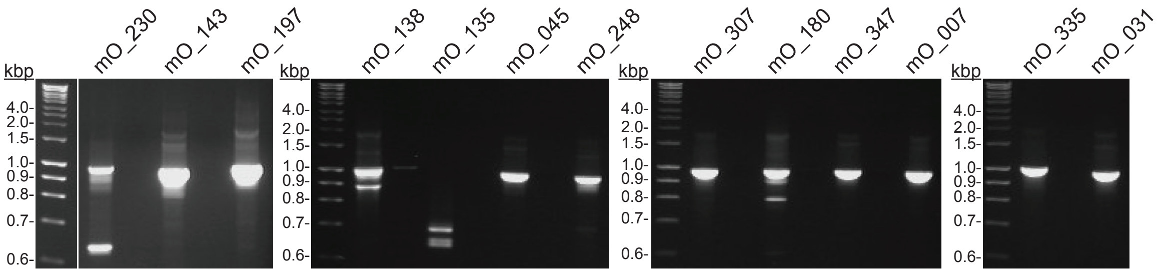

Supplementary Figure S3. Confirmation of minicircles assembled from Illumina reads by PCR. Primers were designed for 13 randomly selected minicircles and used in PCR reactions using pooled DNA samples from Table 1. Predicted amplicon sizes were as follows: minicircle 230 (mO_230): 1,048 bp; mO_143: 988 bp; mO_197: 1,054 bp; mO_138: 1,020 bp; mO_135: 1,016 bp; mO_045: 997 bp; mO_248: 983 bp; mO_307: 972 bp; mO_180: 993 bp; mO_347: 988 bp; mO_007: 973 bp; mO_335: 1,033 bp; mO_031: 985 bp. Only the primers designed for mO_135 failed to produce an amplicon of the predicted size (no attempts were made to optimize the PCR conditions). Amplicons for the other 12 products were excised from the gel and either sequenced directly or after cloning into a plasmid. All matched the expected sequence.

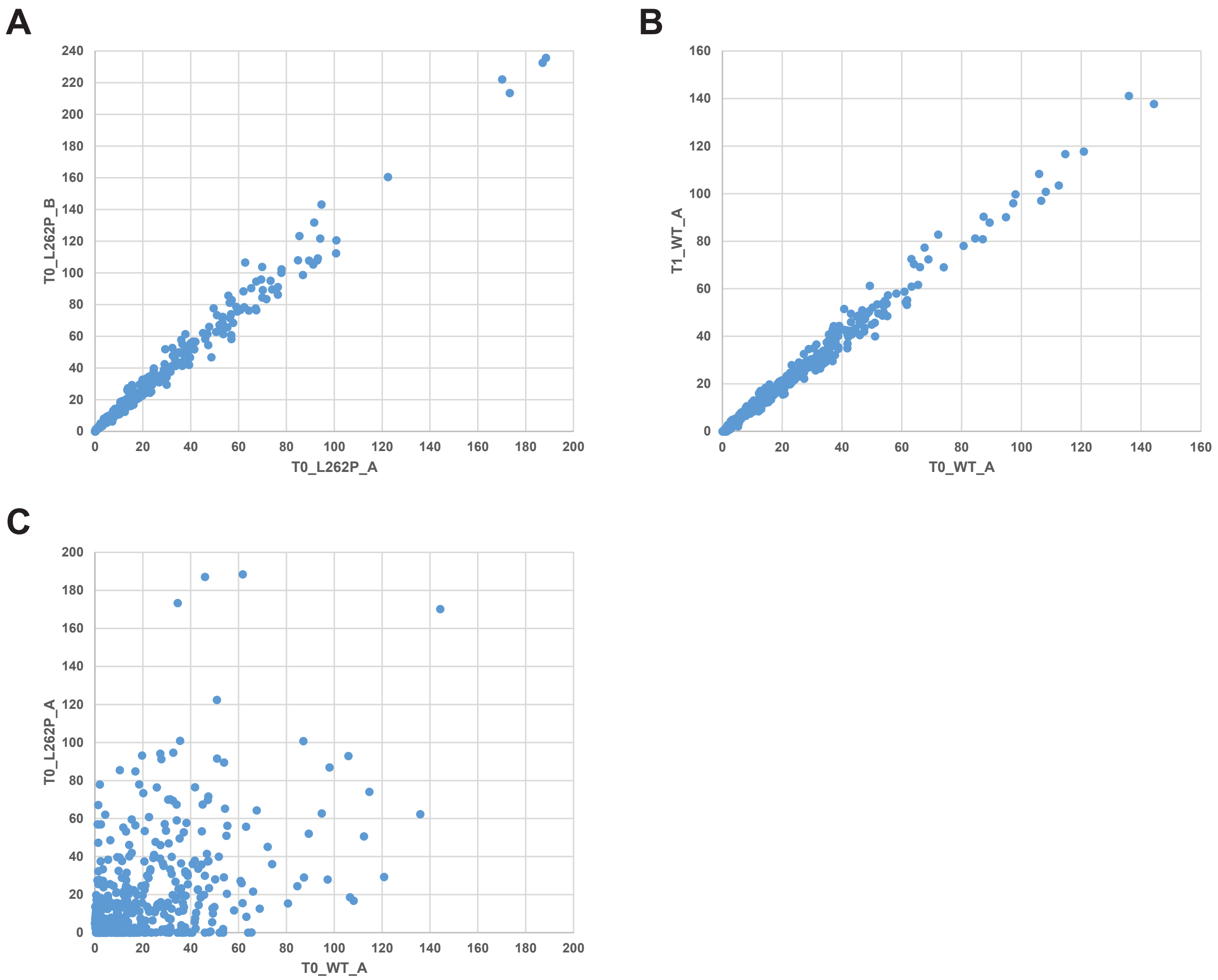

Supplementary Figure S4. Copy number comparisons between samples. Numbers on both axes on the graphs indicate average copy number. Minicircle classes were ordered along the x-axis according to increasing copy number in the sample named on that axis. **(A)** T0_L262P_A vs. T0_L262P_B, **(B)** T0_WT_A vs. T1_WT_A, **(C)** T0_WT_A vs. T0_L262P_A.

A

```
A6pre        G  A  A A  G  G  GAAA   GUUGU   G  AUUUUGGAGUUAUAGAAUAAGAUCAAAUAAGUUAAUAAUA
A6ed     762 GuuAuuAuAuuGuuGuuGAAAuuuG  GUuu-GuuAUU  GGAGUUAUAGAAUAAGAUCAAAUAAGUUAAUAAUA 820
             |||||||||||||||||||||||||  |||  || |||  |||||||||||||||||||||||||||||||||||
A6ed_v1  762 GuuAuuAuAuuGuuGuuGAAAuuuG  GUu--Gu-AUU  GGAGUUAUAGAAUAAGAUCAAAUAAGUUAAUAAUA 818
             :||||:|||||:|:::|||||:|||  :|:  | :|||  ||||||||||.||
          ...UAAUAGUAUAAUAGUGACUUUGAAC  UAG--CG-UAA  CCUCAAUAUCAUA mO_345(II)_gA6_v1(750-793)
             |||||||||||||||| |||| |||  |||  || |||  |||||||||||||||||||||||||||||||||||
A6ed_v2  762 GuuAuuAuAuuGuuGu-GAAA-uuG  GUuuuGuuAUU  GGAGUUAUAGAAUAAGAUCAAAUAAGUUAAUAAUA 819
             :||||:||||:::||| :||| :||  :|||:||:|||  ||||||||||.||
          ...UAAUAGUAUAGUGACA-UUUU-GAC  UAAAGCAGUAA  CCUCAAUAUCAUA mO_229(II)_gA6_v2(746-794)
```

B

```
ND8pre       G     G A GCA G   GCCCGACAGAUUUUUGCCA  ACGCAUUCA  G   G  A G G     G  G   A
ND8ed    351 GuuuuuGuAuGCAuGuuuGCCCGACAGAU    GCCAuuACGCAUUCAuuGuuuGuuAuGuGuuuuuGuuGuuuA 421
             |||||||||||||||||||||||||||||    ||||||||||||||||||||||||||||||||||||||||||
ND8ed_v1 351 GuuuuuGuAuGCAuGuuuGCCCGACAGAU    GCCAuuACGCAUUCAuuGuuuGuuAuGuGuuuuuGuuGuuuA 421
                 |::|||:|||:::|:|.|||||:||    :||||||||..|...||
                 AGUAUAUGUAUGGAUGUGCUGUUUA    UGGUAAUGCUAAUUAAA mO_208(IV)_gND8_v1(355-388)
             ||||||||||||||||||||||||||||     ||||||||||||||||||||||||||||||||||||||||||
ND8ed_v2 351 GuuuuuGuAuGCAuGuuuGCCCGACAGA-    GCCAuuACGCAUUCAuuGuuuGuuAuGuGuuuuuGuuGuuuA 420
                 ||:||::|||:|:|:|.|||||:|     ||||:|||||||:|||...|
                 AAUAUGUGUAUAGAUGAGCUGUUU-    CGGUGAUGCGUAGGUAUAUA mO_365(V)_gND8_v2(355-390)

ND8       97   F  C  M  H  V  C  P  T  D      A  I  T  H  S  L  F  V  M  C  F  C  C  L   119
ND8v1     97   F  C  M  H  V  C  P  T  D      A  I  T  H  S  L  F  V  M  C  F  C  C  L   119
ND8v2     97   F  C  M  H  V  C  P  T  E       P  L  R  I  H  C  L  L  C  V  F  V  V  *  118
```

C

```
ND8pre         GG  G  G    A G  A   GA     A   G G    G G AG  A    A    GG G GA   A  GUG
ND8ed    452 uuGGuuGuuGuuuuAuGuuAuuuGAuuuuuAuuuGuGuuuuGuGuAGuuAuuu-AuuuuGG-GuGAuuuAuuGUG 528
             ||||||||||||||||||||||||||||||||||||||||||||||||||||| ||||||| |||||||||||||
ND8ed_v1 452 uuGGuuGuuGuuuuAuGuuAuuuGAuuuuuAuuuGuGuuuuGuGuAGuuAuuu-AuuuuGG-GuGAuuuAuuGUG 528
                  |:|:::|||||::|||:|||:|:||||:|:||||:|||||||||||.| ||
                  AUAGUGAAAUAUGAUAGACUGAGAAUAGAUACAAGACACAUCAAUAUA-UA mO_134(IV)_gND8(461-506)
             ||||||||||||||||||||||||||||||||||||||||||||||||||||| ||   || |||||||||||||
ND8ed_v2 452 uuGGuuGuuGuuuuAuGuuAuuuGAuuuuuAuuuGuGuuuuGuGuAGuuAuuuuAu---GGuGuGAuuuAuuGUG 526
                  |:|:::|||||::|||:|||:|:||||:|:||||:|||||||||||.|..
                  AUAGUGAAAUAUGAUAGACUGAGAAUAGAUACAAGACACAUCAAUAUAUA mO_134(IV)_gND8(461-506)
                                                 :|:|::|:||::|||:|||:   :|||||||:|||||||
                                                 UAGAGUAUAUUGAUAGAAUG---UCACACUAGAUAACAC
                                                                               |.|||:|::
                                                                               UUAAUGAUG

ND8      132   G  C  C  F  M  L  F  D  F  Y  L  C  F  V  * 145
ND8v1    132   G  C  C  F  M  L  F  D  F  Y  L  C  F  V  * 145
```

```
ND8pre          A G A   AAUUUAGAAUUA  C AUGGUGAAAUUAAAUUUUGACUAAAU
ND8ed    529 uuuAuG-AuuuAA   AGAA  AuuC-ACGGUGAAAUUAAAUUUUGACUAAAU 574
             |||||| ||||||   ||||  |||| |.||||||||||||||||||||||||
ND8ed_v1 529 uuuAuGuAuuuAA   AGAA  AuuCuAUGGUGAAAUUAAAUUUUGACUAAAU 576
             |||||||||||||   ||||  |||||||||||||||||||||||||||||||
ND8ed_v2 527 uuuAuGuAuuuAA   AGAA  AuuCuAUGGUGAAAUUAAAUUUUGACUAAAU 574
             |||||:|||.|
             AAAUAUAUAUA mO_033(II)_gND8_v2(491-531)
             |:|||::||:|||   ||||  |:|||||||||||||||.||.|
             AGAUAUGUAGAUU   UCUU  UGAGAUACCACUUUAAUAUAUA mO_004(V)_gND8(519-562)
```

Supplementary Figure S5. Sequence alignments of the two different edited versions of **(A)** A6 and **(B, C)** ND8 mRNAs that were used for identification of canonical gRNAs. Some key gRNAs are also shown (in blue). The region of A6 affected by the differences is downstream of the predicted stop codon. For ND8, the predicted translation product is indicated, as the additional deletion in ND8 v2 of a U at position 379 would change the reading frame and result in a truncated protein. Also shown are the published sequences (A6 = GenBank accession number M33228; ND8 = GenBank accession number M63820) and the pre-edited mRNA sequences. Differences to the published sequences are shown in red.

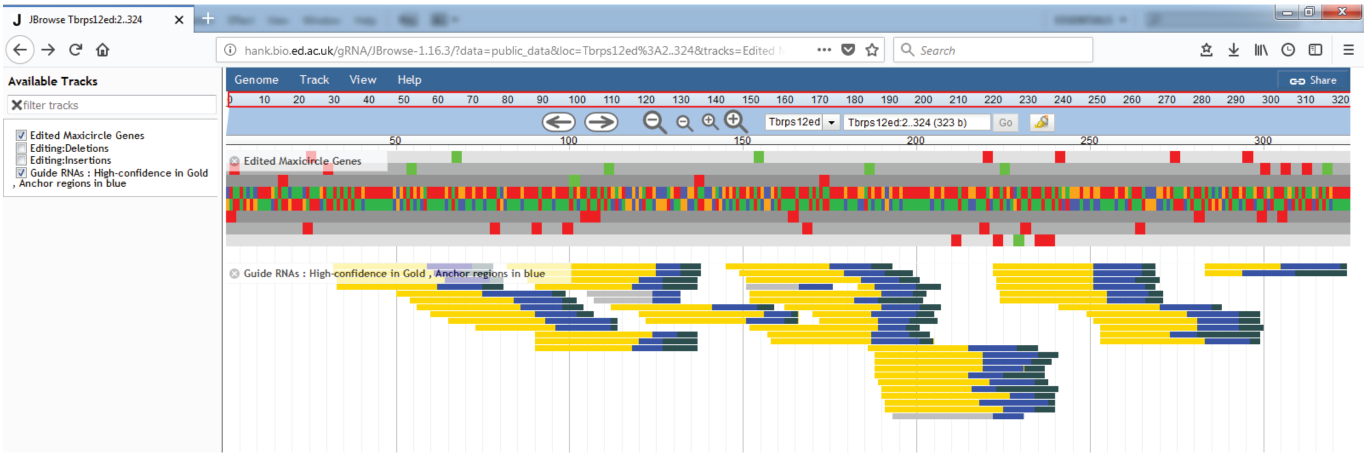

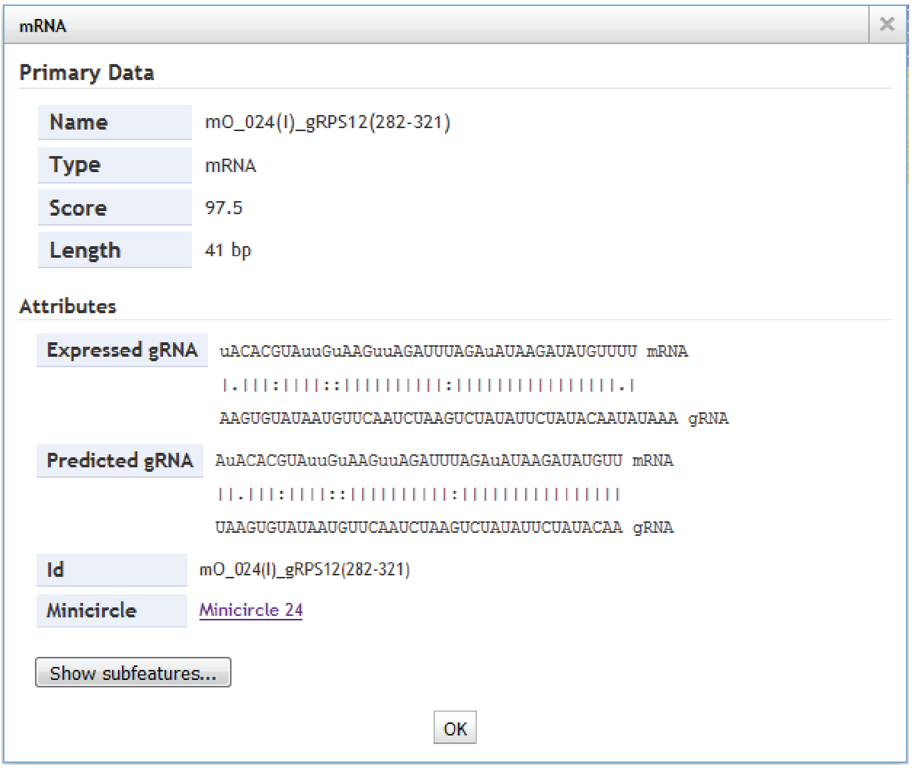

Supplementary Figure S6. Example of gRNA-mRNA alignments available on http://hank.bio.ed.ac.uk, implemented via JBrowse (Buels et al., 2016). Shown is RPS12 as an example. The mRNA of interest can be selected from the pull-down menu. The magnification level, down to single nucleotide resolution, can be controlled by buttons; shown is the most zoomed out level. At this level of magnification, the mRNA sequence of the forward and reverse strands is displayed as coloured boxes, representing the four nucleotides (A = green; C = blue; G = orange; T = red). Start and stop codons for the six open reading frames are shown in green and red, respectively, above and below the mRNA sequence. All predicted gRNAs are shown below the mRNA, with anchor regions in blue and unpaired 5' ends in dark grey. Information regions of high confidence gRNAs (i.e. those confirmed by transcriptome analysis) are shown in gold, information regions of other gRNAs are in light grey. Clicking on a gRNA (in this case the initiator gRNA) displays a pop-up window that shows information such as the name of the gRNA, the score of its match to this mRNA, the length of the alignment, and a link to the minicircle (in GenBank format) that encodes this gRNA.

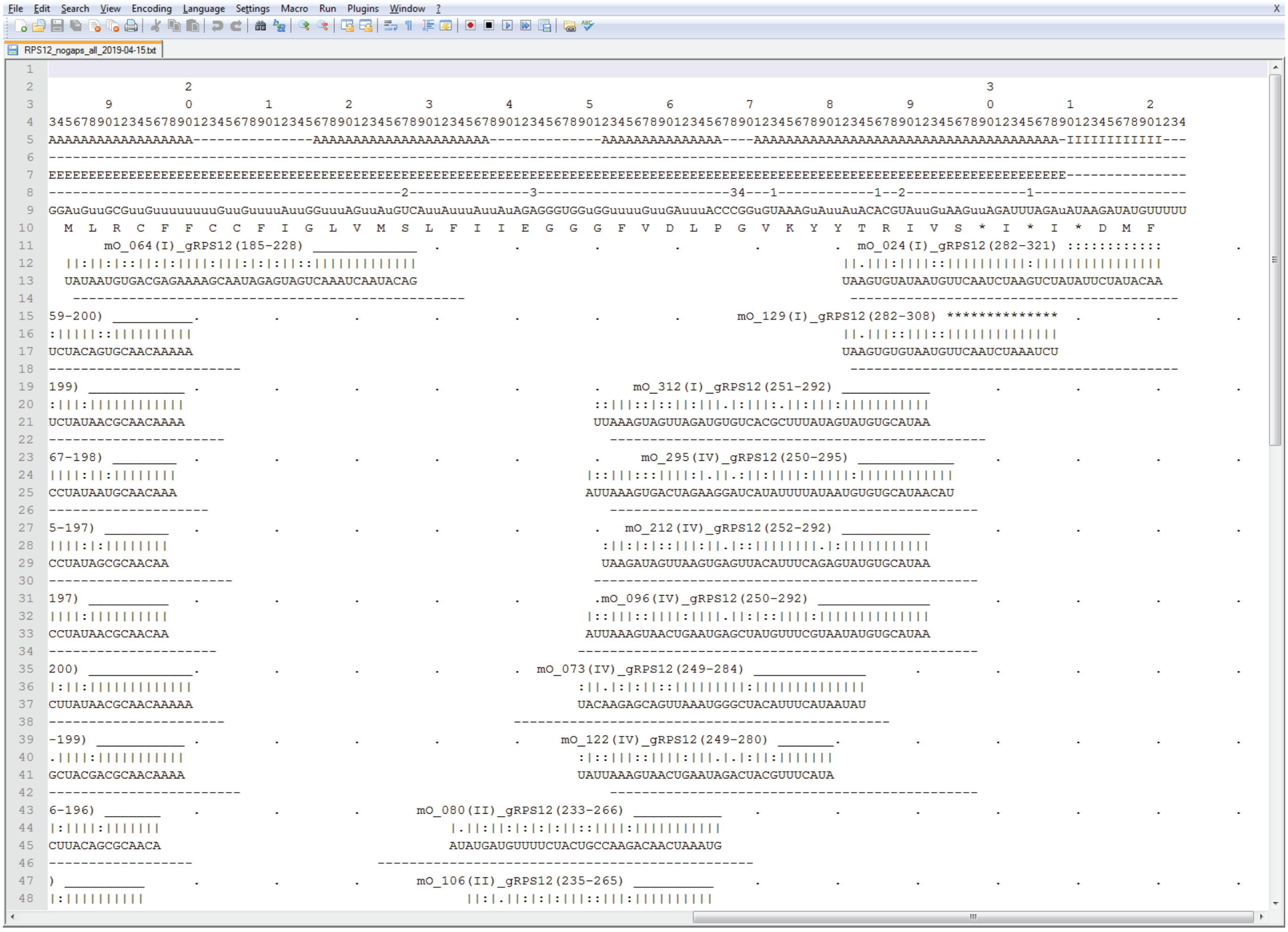

Supplementary Figure S7. Example for the gRNA-mRNA alignments available as plain text files, viewed in Notepad++ (available for free from https://notepad-plus-plus.org). Note that the standard Notepad application provide with Microsoft Windows is not suitable for viewing these files. For Mac users, a suitable viewer is Brackets (available for free from http://brackets.io). Shown as example is the 3' end of the RPS12 mRNA.

Lines 1-4: mRNA position (thousands, hundreds, tens, units) starting from 1.
Line 5: "A"s represent positions on the mRNA at which extender gRNAs anchor; "I"s represent positions on the mRNA at which initiator gRNAs anchor; "U"s represent positions on the mRNA at which extender gRNAs anchor but no 3' gRNA exists which edits the anchor.
Line 6: "M"s represent positions of U-insertions not covered by gRNAs.
Line 7: "E"s represent positions on the mRNA covered by expressed gRNA editing regions.
Line 8: number of deletions to the right of the nucleotide below.
Line 9: edited mRNA sequence 5' to 3' (lowercase "u"s represent insertions).
Line 10: protein sequence.

For each gRNA:
Line 1: name (mO_name(cassette position)_mRNA(start-end of alignment on mRNA)); anchor represented by: "-" for extender gRNA; ":" for initiator gRNA; '.' for unanchored gRNA; '*' for undetermined extender or initiator.
Line 2: base-pairing: "|" Watson-Crick basepair; ":" GU basepair; "." mismatch basepair; "-" gap (only in gapped dataset)
Line 3: gRNA sequence 3' to 5'
Line 4: extent of corresponding small RNA sequence from transcriptome data

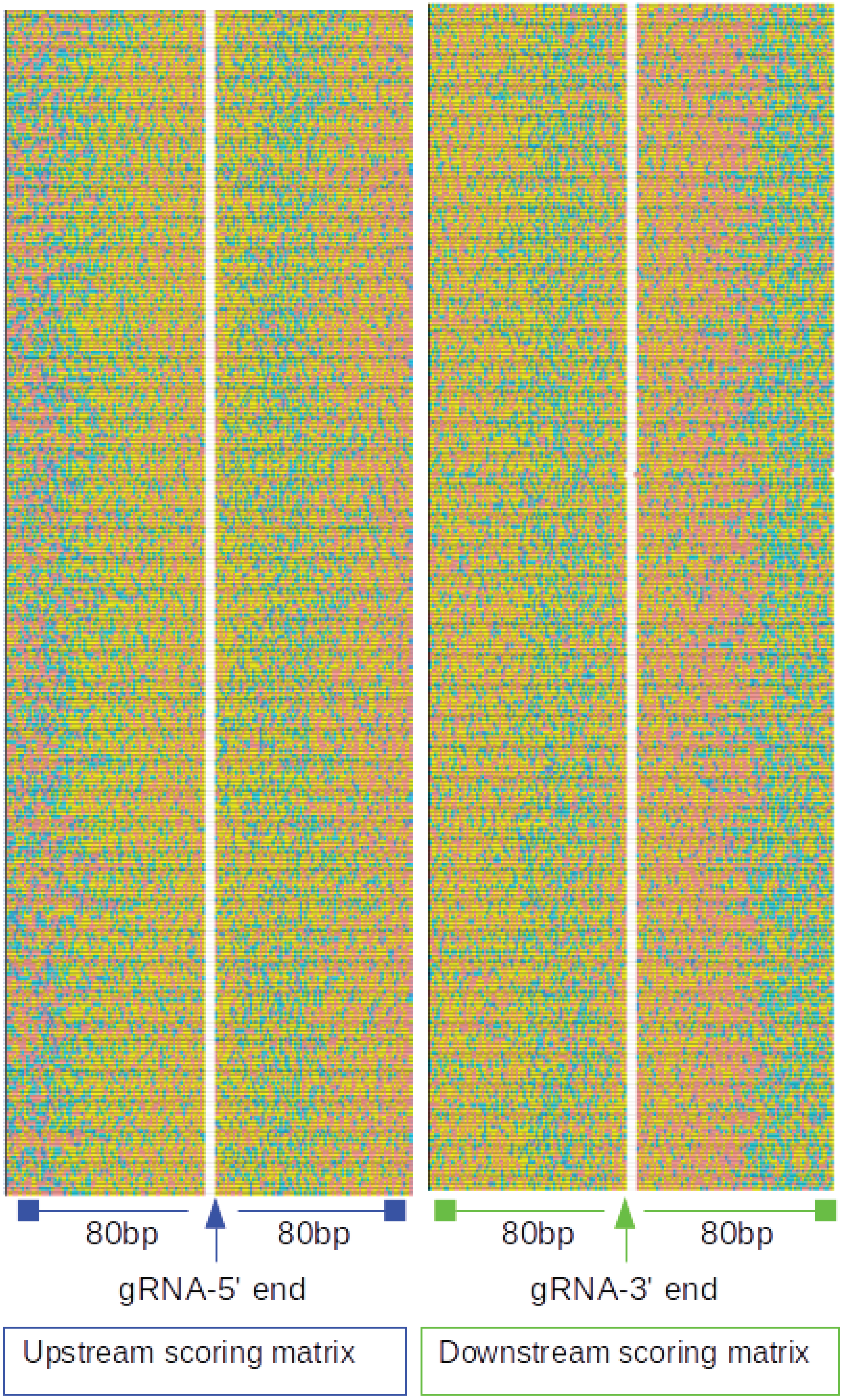

Supplementary Figure S8. Nucleotide bias of regions upstream and downstream of gRNA genes predicted by alignment to edited mRNAs. Guide RNAs were aligned at their 5' ends. Sequences from -80 upstream to +80 downstream of the 5' ends and 3' ends of the aligned gRNA genes were plotted in the Belvu alignment viewer (Sonnhammer and Hollich, 2005); the colours are as follows: T, red; A, yellow; G, green; C, blue. The 5' and 3' ends of the predicted gRNA-mRNA duplexes are indicated.

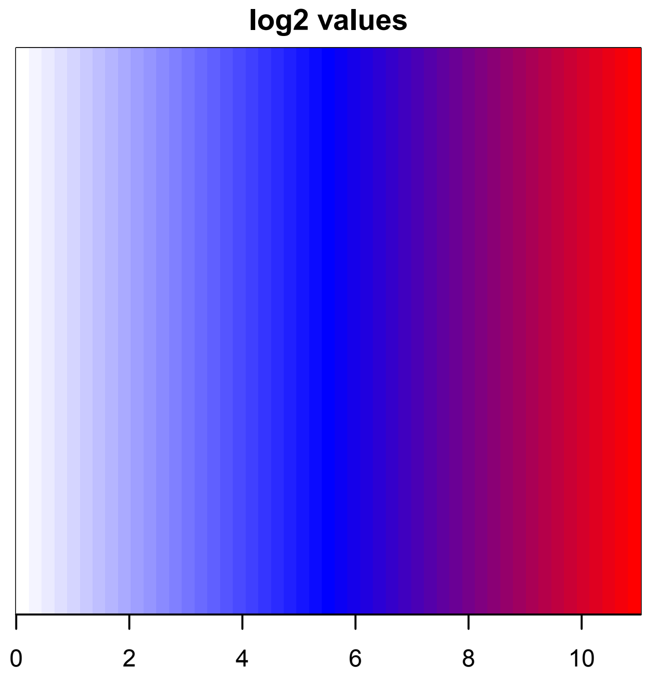

Supplementary Figure S9. 'All vs. all' BLAST analysis of all 391 minicircles, using wordsize = 24 Nucleotides 1-100 (encompassing CSB-1, CSB-2 and CSB-3; nucleotide 1 corresponds to the first nucleotide of the CSB-1 motif, see Supplementary Figure S2) were trimmed off for this analysis. Colours in the heatmap correspond to log2 values of the HSP score. The 50 most related minicircle pairs are shown in Supplementary Table S3, along with the scores (log2-transformed and untransformed) for this 'all vs. all' BLAST analysis, scores for individual BLAST analyses, and percent identity values from pairwise alignments with EMBOSS matcher (https://www.ebi.ac.uk/Tools/psa/emboss_matcher/).

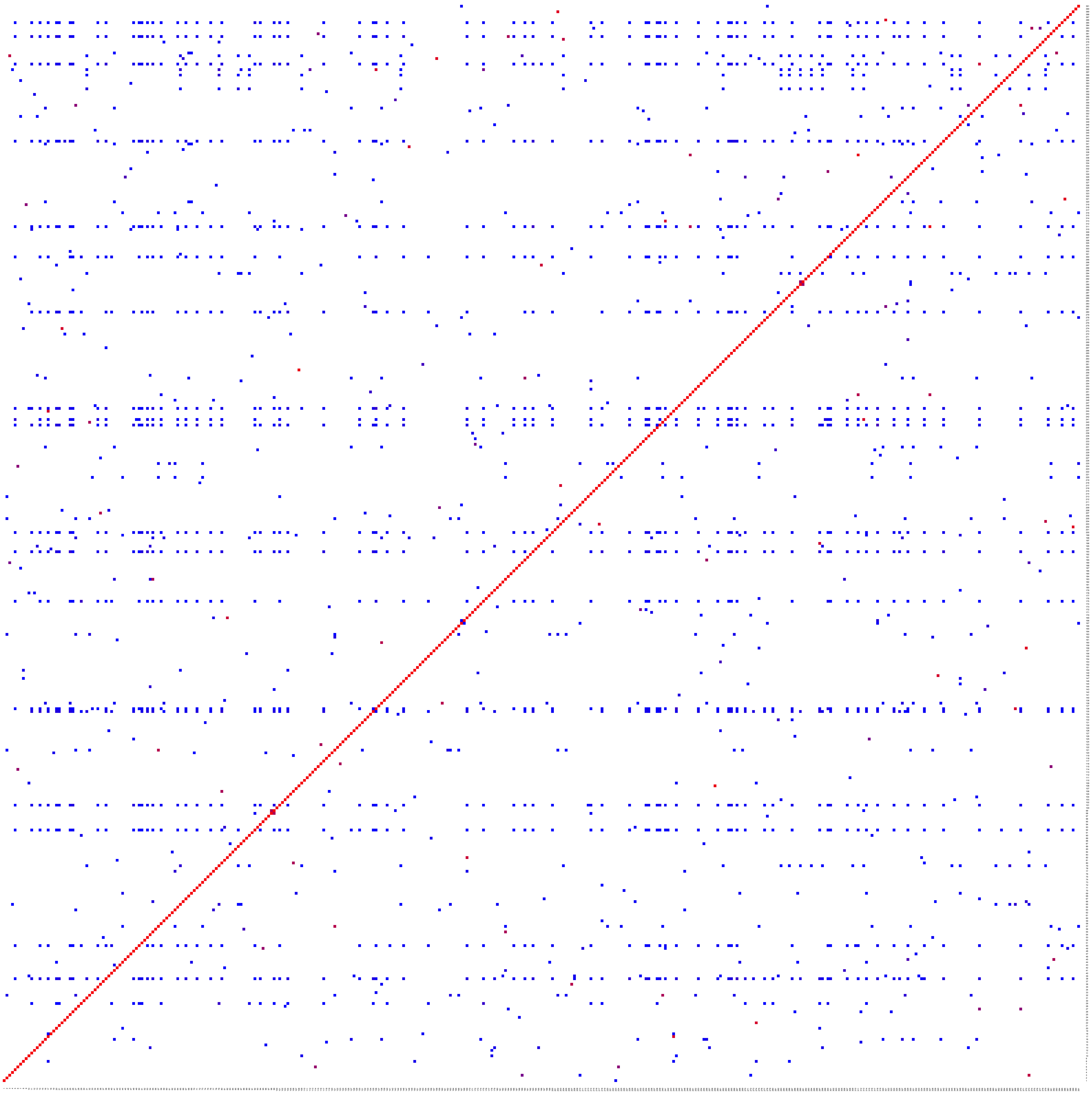

```
                 CSB-1                     CSB-2
mO_108       1 GGGCGTGCAGATTTCACCATACACAAATCCCGTGCTATTTTAGGGGGGTTTTGAGGTCGGAGGTACTTCGAAAGG     75
               |||||||||.|..|...||||||||||||||||||||||||.|||...||||.|||||.||||||||||||||||
mO_259       1 GGGCGTGCAAAAATACACATACACAAATCCCGTGCTATTTTGGGGCATTTTTTAGGTCCGAGGTACTTCGAAAGG     75
                 CSB-1                     CSB-2
                  CSB-3
mO_108      76 GGTTGGTGTAATACTCACACGGTTTTCGAGGACTTTTTCAGGGTTTTCGGAGTTACTGGGTTACTGGGTTACTGG    150
               ||||||||||||||.||||.||||||||.||||||||||||||||||.||.||||.||| |||.||||||||||.
mO_259      76 GGTTGGTGTAATACACACAGGGTTTTCGGGGACTTTTTCAGGGTTTTTGGGGTTATTGG-TTATTGGGTTACTGA    149
                  CSB-3
                                      fwd-rpt
mO_108     151 GTGTTTGGGCTTAGAAAGGGAAATGGAATAATAGATAAATTATAATATTTAATATATAGAGTTAGTATACATATA    225
               |||||.|||||||||||||||||||||||||||||||.|||||||||||||||||||||||||||||||||||||
mO_259     150 GTGTTCGGGCTTAGAAAGGGAAATGGAATAATAGATAGATTATAATATTTAATATATAGAGTTAGTATACATATA    224
                                      fwd-rpt
                gRNA predicted by nt bias                                          rev-rpt
mO_108     226 AATAACATGCAAGCTATCGTAACTGATTTTACAGAGATAATAAGTATATATAATTAAGTTATAAATCTAGTTATT    300
               |||||||||||||||||||||||||||||||||||||||||||||||||||||||||||||||||||||||||||
mO_259     225 AATAACATGCAAGCTATCGTAACTGATTTTACAGAGATAATAAGTATATATAATTAAGTTATAAATCTAGTTATT    299
                gRNA predicted by nt bias                                          rev-rpt

mO_108     301 ATATTATTTATTTTTAGTCAGAGGGGTAAGGTATAGATTGGTGAAGCGAGGTAATTGTTTTCTGTGATTTCGGTG    375
               |||||||||||||| |||||.||.|.||||||||||||||||||.||.|||||||||||||||||||||||||||
mO_259     300 ATATTATTTATTTT-AGTCAAAGAGATAAGGTATAGATTGGTGAGGCAAGGTAATTGTTTTCTGTGATTTCGGTG    373

                          fwd-rpt
mO_108     376 GAGGAGAAAGTAAGGTAATAGATAGAAATAGAAAGTTAATACTTAATAATATGTATATATAACTATAACGAAACA    450
               |||||||||.||||.||||||||||.||||.||||||||||.|||||||||||||||||||||||||||||||||
mO_259     374 GAGGAGAAAATAAGATAATAGATAGGAATATAAAGTTAATAATTAATAATATGTATATATAACTATAACGAAACA    448
                          fwd-rpt
               gRNA predicted by nt bias                                    rev-rpt
mO_108     451 GATA-GATGTACCTATATAGATAAATTATAATTATGTTATATTAAAGTTAATTACTTATCTATTATTGTATTTTA    524
               |||| ||||||||||||||||||||||||||||||||||||||||||||||||||||||||||||||||||||||
mO_259     449 GATAAGATGTACCTATATAGATAAATTATAATTATGTTATATTAAAGTTAATTACTTATCTATTATTGTATTTTA    523
               gCR4(137-182) predicted by alignment                         rev-rpt
                                                                   fwd-rpt
mO_108     525 A-TGGTGGAATGCTGTGAGTAGTAGTATAATTGGGCTTAGAGAGGCTTACTAGGTAAAAGAAATGGGATAATAGA    598
               | |||||||||||||....||||||||||||||.||||||||||.||..||||||||||||||||||||||||||
mO_259     524 AATGGTGGAATGCTGCAGATAGTAGTATAATTGAGCTTAGAGAGACTCTCTAGGTAAAAGAAATGGGATAATAGA    598
                                                                   fwd-rpt
                                                       gA6(287-340) predicted by alignment
mO_108     599 TAAAGGTATAGATAATTAATATAGTATAGATAATATAAACAAAACAAAGATAAAGATGCAGTATATAGTAGAGAA    673
               ||||||||||||||||||||||||||||||||||||||||||||||||||||||||||||||||||||||||||.
mO_259     599 TAAAGGTATAGATAATTAATATAGTATAGATAATATAAACAAAACAAAGATAAAGATGCAGTATATAGTAGAGAG    673
                                                       gA6(292-340) predicted by alignment
                                                     rev-rpt                       fwd-rpt
mO_108     674 ATGGTATTAAGTAATTATATTTATTTTGTAACTTATTTATTATATTATTTGTATTTCTAGATGGGTGGATTGGAT    748
               |||.||||||.||||||||||||||||||||||||||||||||||||||||||||..|||||.||||
mO_259     674 ATGATATTAATTAATTATATTTATTTTGTAACTTATTTATTATATTATTTGTATTTCTAGATGAATGGATAGGAT    748
                                                     rev-rpt                       fwd-rpt
                                                                 gRNA predicted by nt bias
mO_108     749 AATAAATAGATGTGAGGTAGGTTAAGTTTAGATATCAGATAATTAATAACGTATGGATTTGATCTAAATGATAAC    823
               ||||||||||||||||.||||||||||||||||||||||||||||||||.|||||.|||||||||||||||||||
mO_259     749 AATAAATAGATGTGAGATAGGTTAAGTTTAGATATCAGATAATTAATAAAGTATGAATTTGATCTAAATGATAAC    823
                                                                 gRNA predicted by nt bias
                                                           rev-rpt
mO_108     824 TGGTAATTTAAGATTTATATAGTAATTAATTTAAGATTTATTTATTACATTATTTTGATAGTAAGTGGTTGGAAG    898
               ||.|||||||||||||||||..|||||||||||||||||||||||||||||||||||.||||||||||||.||||
mO_259     824 TGATAATTTAAGATTTATATGATAATTAATTTAAGATTTATTTATTACATTATTTTGTTAGTAAGTGGTTAGAAG    898
                                                           rev-rpt

mO_108     899 AGATTCAAATAGAGAATTAATAGTTATGTGGGAGTGGTATTGTGAGGA-TAAAATTGTCAAAAAATCAAGAAAAA    947
               |||||||||||||||||||||||||||||||||||||||||||||||| ||||||||||||||||||||||||||
mO_259     899 AGATTCAAATAGAGAATTAATAGTTATGTGGGAGTGGTATTGTGAGGAATAAAATTGTCAAAAAATCAAGAAAAA    948


mO_108     948 TGGGCAAAAATCACCTCGAAAAAACCGAAAATCTTA   1008
               |||||||||||.||||||||||| |.||||||||||
mO_259     949 TGGGCAAAAATTACCTCGAAAAA-CTGAAAATCTTA   1008
```

Supplementary Figure S10. Alignment of minicircles mO_108 and mO_259, generated with EMBOSS matcher (https://www.ebi.ac.uk/Tools/psa/emboss_matcher/). Note the region annotated as 'gRNA predicted by nucleotide bias', i.e. a non-canonical gRNA, in mO_108 (position 436-474) and as 'gCR4(137-182)' in mO_259 (position 434-479). A gap in the alignment between the two minicircles in this region (indicated by a red arrow) corresponds to a gap in the alignment between this region of mO_108 and mRNA CR4. This prevents identification of this region as gCR4(137-182) in the more stringent of our pipelines as it requires non-gapped alignments. Conserved sequence blocks CSB-1 to 3 are indicated, as are the inverted 18-bp repeats (fwd-rpt, rev-rpt).

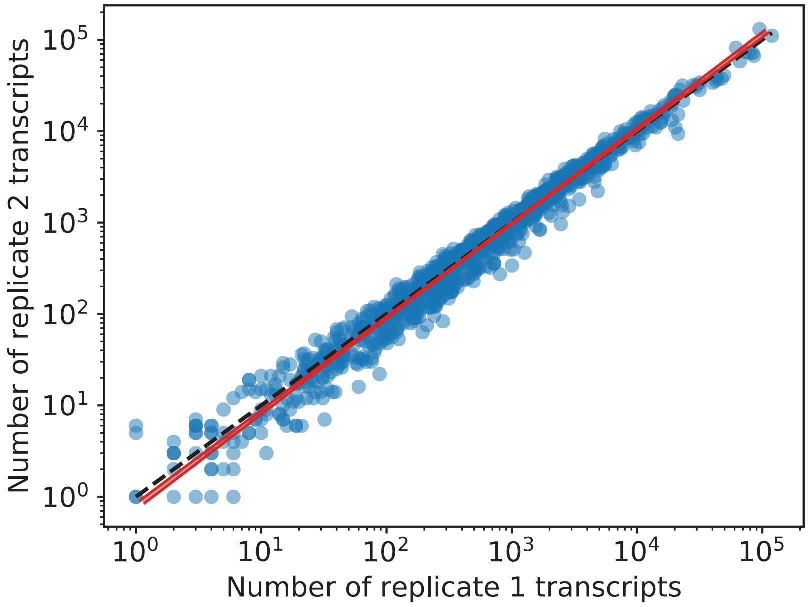

Supplementary Figure S11. Transcript abundances in each cassette (1295 in total) are highly correlated between the two replicates. For each replicate, samples for BSF and PCF were combined and mapped to the 391 minicircles. Each blue circle in the plot represents a particular cassette. The black dashed line is y=x, i.e., perfect correlation. The red region shows the orthogonal distance regression with 95% CIs.

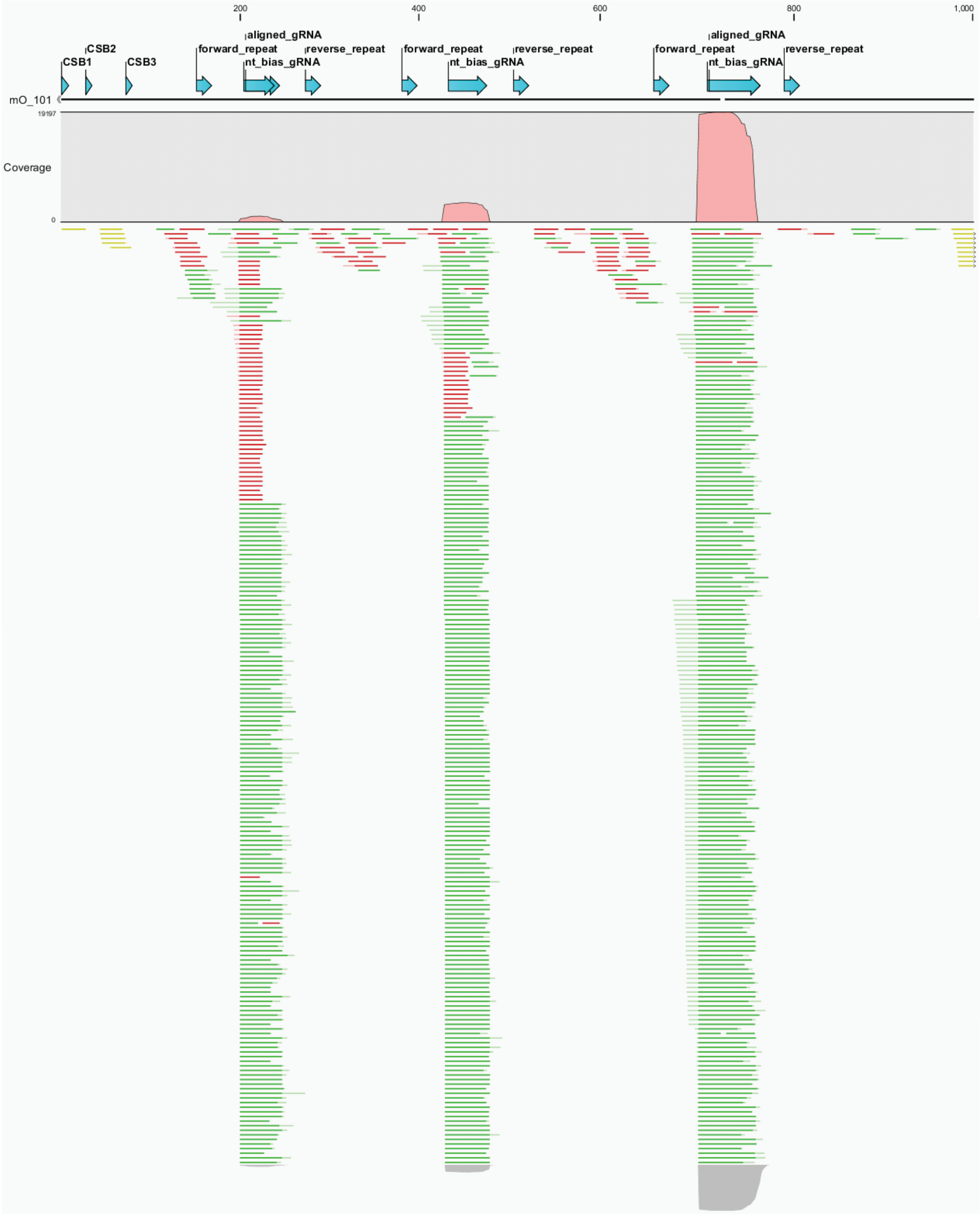

Supplementary Figure S12. Mapping of non-nuclear, adapter-trimmed small RNAs (combined BSF and PCF reads, replicate 2) to minicircle mO_101 (mapped and visualised in CLC Genomics Workbench). Reads mapping to the top and bottom strands are shown in green and red, respectively; yellow colour indicates reads that mapped with the same score to more than one location in the minicircle population. Terminal regions of read sequences that did not align to the minicircle sequence (e.g. 3' oligo-(U) tails) are indicated by a lighter shade. 'Overflow' reads that could not be displayed due to space limitations are indicated by the grey region on the bottom. CSB motifs, 18-bp inverted repeats, and gRNA genes predicted by alignment to known edited mRNAs (aligned_gRNA) and nucleotide bias (nt_bias_gRNA) are indicated (compare Supplementary Figure S13).

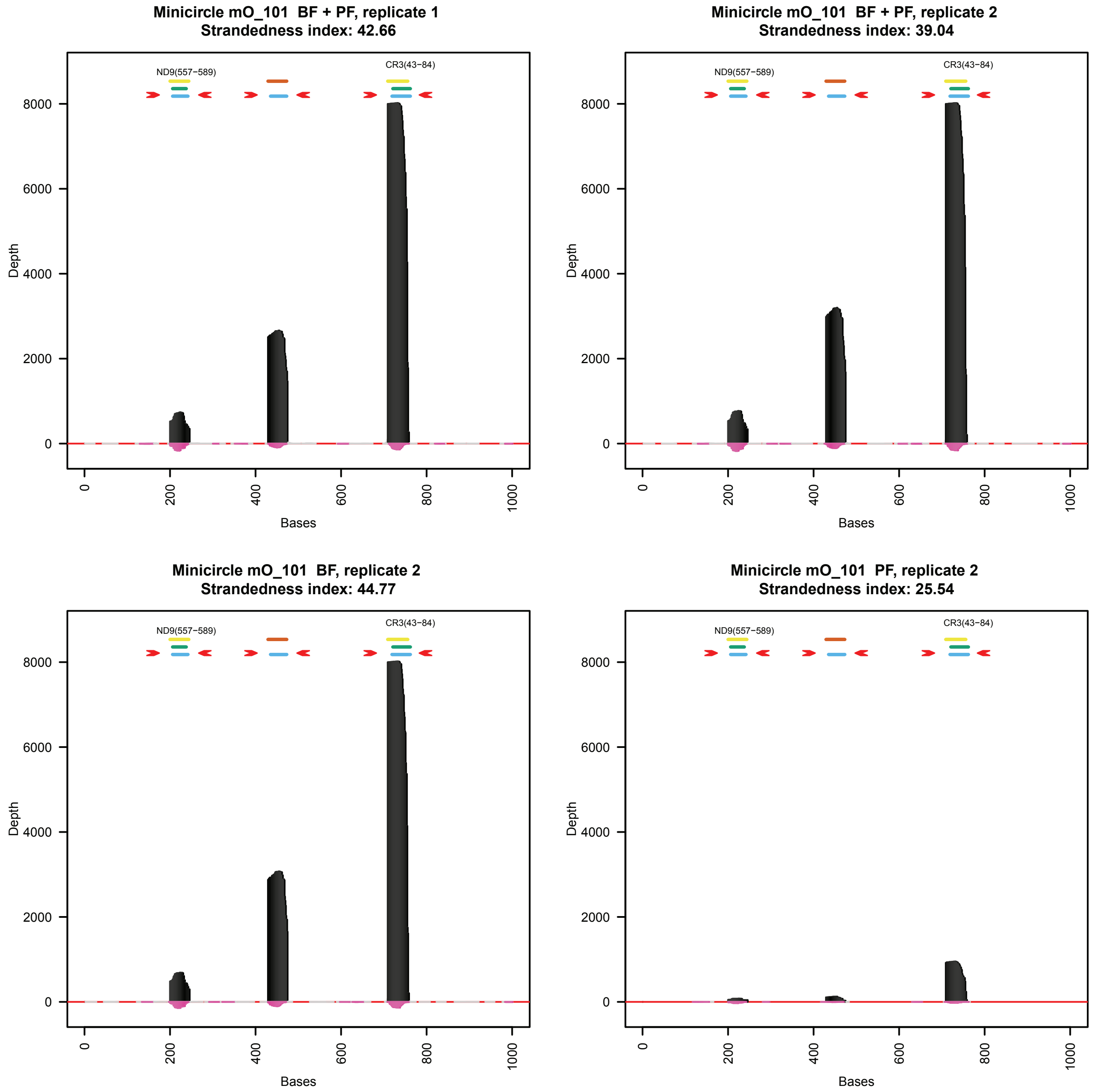

Supplementary Figure S13. Analysis of minicircle coverage with short sense and antisense transcripts, exemplified by minicircle mO_101 (data for all 391 minicircles are available on Figshare, https://doi.org/10.6084/m9.figshare.7756808.v1). Regions within each depth plot considered as being expressed were identified as regions where read depth exceeded a subjectively assigned percentage of 0.025% of total read depth. These are indicated by black bars for sense and by pink bars for antisense reads. Depths not exceeding the threshold are shown in grey. Transcript populations mapping to minicircle sequence and gRNA gene annotations in the minicircle are identified by coloured bars: yellow, short transcript aligning to a known (canonical) edited mRNA; orange, short transcript not aligning to a known edited mRNA (non-canonical); blue, gRNA gene predicted by nucleotide bias; green, canonical gRNA gene predicted by alignment to a known edited mRNA. Forward and reverse 18-bp repeats are indicated by red arrowheads.

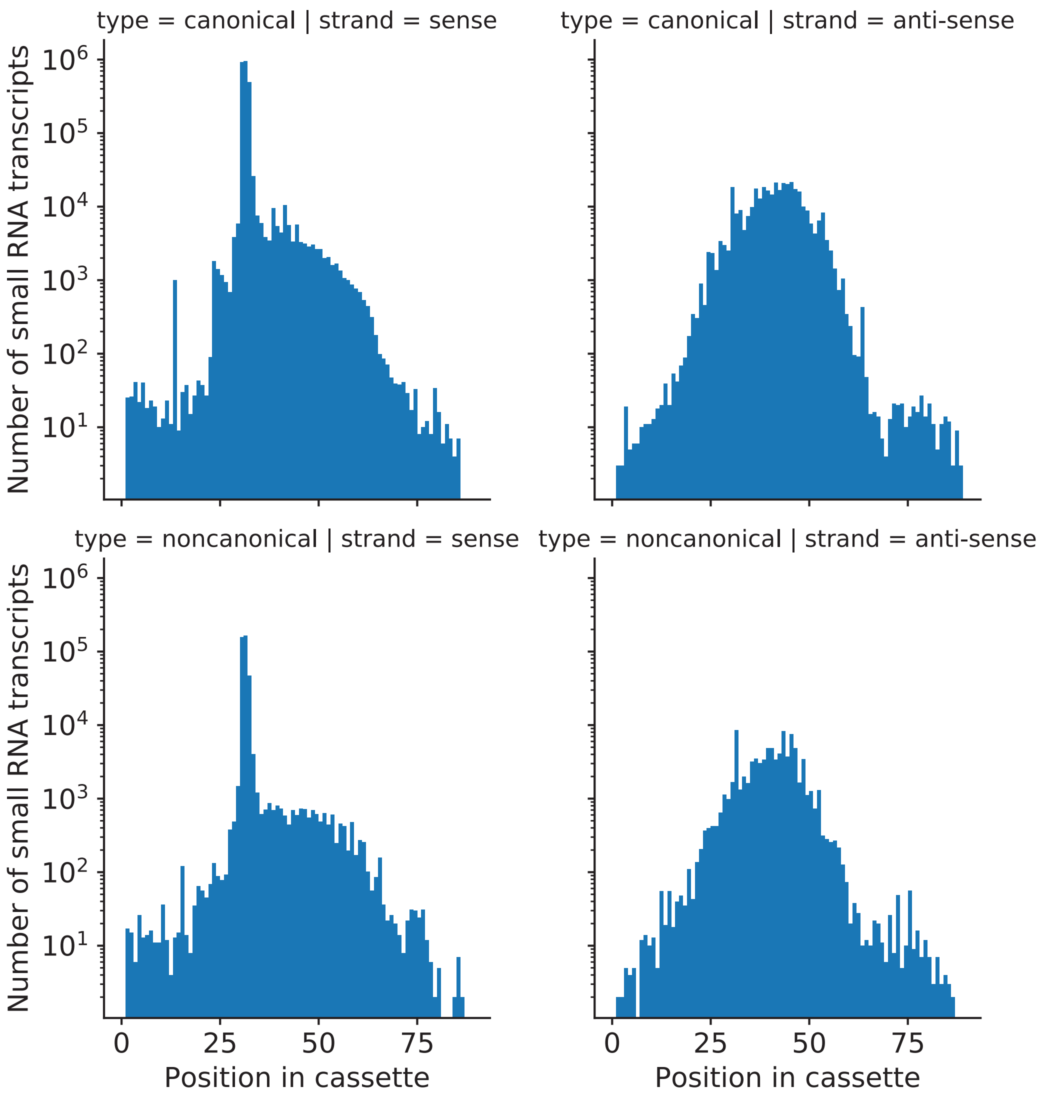

Supplementary Figure S14. The distance of small RNA transcript 5′ ends relative to the 18-bp repeats. For sense transcripts (i.e. canonical and non-canonical gRNAs), the distance given is the distance from the 3' end of the forward 18-bp inverted repeat to the position on the coding strand that corresponds to the 5' end of the transcript. For antisense transcripts, the distance given is the distance from the nucleotide on the minicircle forward strand (template strand for antisense-transcripts) that corresponds to the 5′ end of the transcript to the 5′ end of the reverse 18-bp inverted repeat. The combined numbers from bloodstream and procyclic form samples are shown. Note the log scale on the y-axis. Most (94.3%) of small RNA sense transcripts in bloodstream and procyclic forms align 30, 31 or 32 nucleotides downstream from the end of the forward 18-bp inverted repeat.

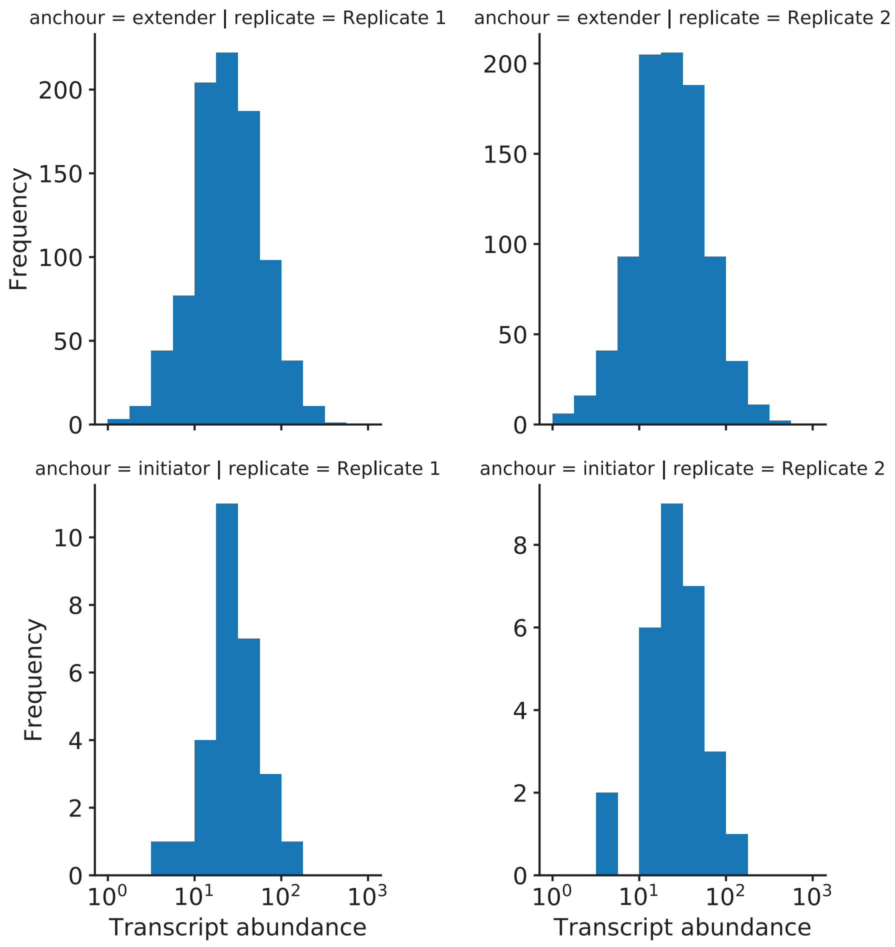

Supplementary Figure S15. Distributions of small RNA transcript abundances of HC canonical gRNAs that extend editing domains (top panels) or initiate editing domains (bottom panels).
